# Balancing photosynthesis and respiration in North Pacific protist communities

**DOI:** 10.1093/ismejo/wrag234

**Published:** 2026-09-11

**Authors:** Sacha N Coesel, Shiri Graff van Creveld, Mathilde Dugenne, Bryndan P Durham, Michael J Follows, Rebecca S Key, Francois Ribalet, Angelicque E White, E Virginia Armbrust

**Affiliations:** School of Oceanography, University of Washington, Seattle, WA 98195, United States; School of Oceanography, University of Washington, Seattle, WA 98195, United States; Department of Oceanography, University of Hawai'i at Manoa, Honolulu, HI 96822, United States; Department of Biology, Genetics Institute, University of Florida, Gainesville, FL 32611, United States; Department of Earth, Atmospheric and Planetary Sciences, Massachusetts Institute of Technology, Cambridge, MA 02139, United States; Department of Biology, Genetics Institute, University of Florida, Gainesville, FL 32611, United States; School of Oceanography, University of Washington, Seattle, WA 98195, United States; Department of Oceanography, University of Hawai'i at Manoa, Honolulu, HI 96822, United States; School of Oceanography, University of Washington, Seattle, WA 98195, United States

**Keywords:** net community productivity, photosynthesis, respiration, mixotrophy, phytoplankton, eukaryotes, zooplankton, metatranscriptome, population size structure, amplicon sequence variance

## Abstract

Marine plankton fuel their metabolism through a continuum of trophic strategies, spanning from pure autotrophy to pure heterotrophy, with mixotrophy combining the two modes within a single organism. Different taxa balance these strategies depending on environmental conditions, complicating efforts to link community composition with ecosystem processes. To quantify the transcriptional investment in processes supporting primary production versus respiration along the trophic continuum, we developed the Transcript-informed Productivity (TiP) metric, defined as the ratio of transcripts in photosynthesis-related pathways (e.g. photosynthesis, carbon fixation) to those in both photosynthesis and respiration-related pathways (e.g. respiration, macromolecule degradation). Higher TiP indicates greater transcriptional investment in primary productivity, whereas lower TiP reflects reduced investment in primary productivity and/or increased investment in catabolic activities. We applied TiP across eukaryotic plankton size classes (~1–100 μm) and taxonomic groups using metatranscriptomes collected during three latitudinal expeditions across the North Pacific. By combining TiP with 18S rRNA gene sequence data and cell size characterization via flow cytometry, we show that biomass in more nutrient-rich areas was dominated by larger phototrophic eukaryotic plankton (diatoms) with higher TiP, consistent with elevated net community productivity (NCP). In nutrient-limited areas, eukaryotic pico- and nano-phytoplankton were dominated by Haptophyta and Dinophyta and displayed reduced TiP, suggesting greater reliance on energy from respiration rather than photosynthesis, consistent with mixotrophic strategies supporting these taxa under nutrient limitation. The TiP metric provided taxon-resolved indicators of eukaryotic productivity and respiration and can complement bulk NCP measurements by identifying the taxa contributing most to ecosystem metabolic balance.

## Graphical Abstract

**Graphical Abstract:**
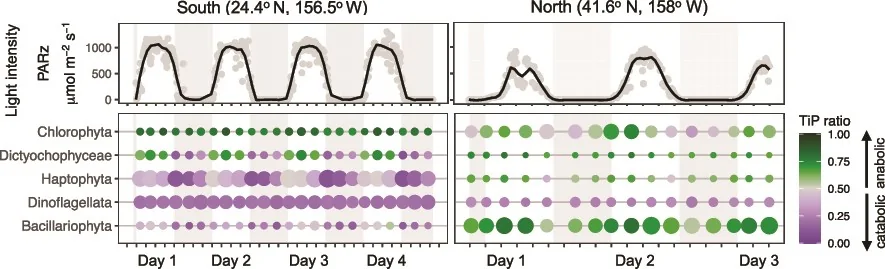

## Introduction

Phytoplankton form the foundation of marine food webs and drive global biogeochemical cycles [1]. Their ecological roles reflect the metabolic diversity that has been shaped by the evolutionary history of photosynthesis in eukaryotes. Ancestral photosynthetic protists were mixotrophic, combining phagocytosis and photosynthesis. A primary endosymbiosis of a cyanobacterium by a heterotrophic protist gave rise to plastids in Chlorophytes and Rhodophytes [2, 3]. Over evolutionary time, repeated plastid gains and losses through secondary and tertiary endosymbioses produced the diversity of plastids seen in taxa such as Stramenopiles, Alveolates, and Haptista. Modern eukaryotic phytoplankton groups include species that display a range of trophic modes and include strictly photosynthetic groups that have lost the ability to phagocytose and mixotrophic groups that display various degrees of photosynthesis and phagotrophy [4, 5]. Furthermore, strictly heterotrophic groups that lost the ability to carry out photosynthesis may nonetheless group phylogenetically with those taxa that retain photosynthetic capabilities [6, 7]. Thus, trophic strategy cannot be inferred from taxonomy alone, yet this trait is central to understanding phytoplankton ecology.

Differential energy requirements associated with the different trophic modes impact phytoplankton cell size and ultimately their biogeography [8, 9]. For instance, small phototrophs such as the cyanobacterium *Prochlorococcus* thrive in nutrient-poor waters due to their high surface area-to-volume ratios and low nutrient requirements, whereas large eukaryotic phototrophs such as diatoms dominate biomass in nutrient-rich environments [10–13]. Eukaryotic phytoplankton in oligotrophic surface waters are predicted to be predominantly mixotrophic [14–16] where they can access nutrients from prey cells to support photosynthesis. And yet, mixotrophs may experience additional cell size constraints, as maintaining both plastids and digestive vacuoles imposes structural demands for larger cells [8, 9]. Because both cell size and trophic strategy strongly influence growth, resource use, and energy transfer, accurately characterizing these traits is essential for understanding plankton dynamics and predicting ecosystem responses to environmental change.

Multiple approaches are used to infer the trophic behavior of eukaryotic phytoplankton groups *in situ*. Laboratory studies of marine isolates inform the behavior of related environmental species, with observations synthesized in the Mixoplankton Database [17, 18] and the Dinoflagellate and Ciliate trophic-mode database [19]. Although powerful, the trophic labels are based on culturable organisms and may not capture natural diversity [20]. Traditional field analyses estimate grazing via microscopy or flow cytometry [21, 22], which are difficult to apply broadly and typically group broad polyphyletic taxa together. More recently, genomic or transcriptomic datasets train machine-learning models to predict trophic modes from metagenomic and metatranscriptomic data [23–25]. And yet, these models do not capture the full continuum of trophic strategies, as they typically impose categorical classifications such as “mixotroph” or “phototroph” [23, 25]. Additional complexity arises from variation within trophic modes, including cases of obligate versus facultative mixotrophy [5, 26].

Rather than focusing on discrete trophic categories, here we quantify the continuum of energy acquisition strategies by assessing transcriptional investment in photosynthetic and respiratory processes. This approach extends a recently proposed framework that correlated the expression levels of carbon fixation and endocytosis/phagocytosis genes [27], by instead focusing on energetic pathways rather than acquisition mechanisms. Transcript-informed Productivity (TiP) measures the ratio of transcripts associated with photosynthesis-related processes (e.g. photosynthesis, carbon fixation) to total photosynthesis and respiration-related transcripts (respiration, organic matter degradation), using curated KEGG Orthology (KO) identifiers [28]. Higher TiP indicates greater transcriptional investment in primary productivity; lower TiP reflects greater investment in respiration-related processes. As such, TiP may help determine whether organisms function as carbon producers, consumers, or are metabolically neutral.

We tracked TiP across taxa and size classes along a North Pacific gradient. Samples were collected during three latitudinal expeditions (Gradients 1–3) spanning the Subtropical Gyre (Gyre) and the Transition Zone (TZ), which separates the North Pacific Subtropical and Subpolar Gyres, along with two high-temporal datasets in the Gyre and TZ. The Gyre and TZ exhibited distinct biogeochemical conditions, allowing examination of links between environmental pressures, population structure, and function [29]. By integrating TiP with cell size measurements from conventional and imaging flow cytometry [30, 31] and 18S rRNA gene sequencing [32], we show that flexibility in balancing photosynthesis and respiration can broaden biogeographic niches. The findings also suggest that eukaryotic phytoplankton taxa may not always function primarily as carbon producers across environmental gradients. Overall, our approach provides a framework for using metatranscriptomes to inform eukaryotic productivity and respiration estimates and better incorporate small- and intermediate-sized mixotrophs into ecological and biogeochemical models.

## Materials and methods

### Cruise transects and sample collection

Samples for metatranscriptome analysis were collected in the North Pacific during spring on Gradients 1, 2, and 3 (G1: KOK1606, April–May 2016; G2: MGL1704, May–June 2017; G3: KM1906, April 2019) along 21–42°N, 158°W, and during the summer on the Diel1 cruise (KM1513; July–August 2015), as described in the North Pacific Eukaryotic Gene Catalog [33]. Samples were collected at 7 m depth using the underway system during G3 and at 15 m depth via CTD rosette for all other datasets. Samples were pre-filtered through 200 μm mesh for G1 and 100 μm mesh for the other cruises. Two size fractions were obtained by sequential filtration onto 3 μm and 0.2 μm filters [33], except for Diel1, which was collected directly onto 0.2 μm filters. Transect sampling occurred pre-dawn at ~4 a.m. (G2), ~5 a.m. (G1), and ~ 6 a.m. (G3). Diel sampling occurred every 4 h for Diel1 (24.4°N, 156.5°W) and G3-diel (41.6°N, 158°W) datasets. Samples were collected in triplicate (duplicate for Diel1), flash-frozen in liquid nitrogen, and stored at −80°C.

Surface temperature, salinity, inorganic nitrogen (nitrate + nitrite), particulate organic carbon (POC), and net community production (NCP) were obtained from Simons CMAP [34]. In short, nitrogen concentrations were measured using a SEAL AutoAnalyzer III [35]. POC was quantified on GF/F filters via high-temperature combustion [31]. NCP was estimated from surface O₂/Ar ratios using equilibrated inlet mass spectrometry, and for Diel1 from 24 h ^14^C primary production incubations corrected for dark uptake [31]. Depth-resolved photosynthetically active radiation (PARz) was calculated as, using *τ* = 0.96 and k_par_ = 0.04 [36], with z corresponding to a sampling depth of 15 m for G1–G2 and 7 m for G3.

### Quantitative metatranscriptome analysis

RNA was extracted, processed, annotated, and published in [33, 37]. Briefly, each sample was spiked with synthetic RNA standards prior to extraction. Polyadenylated mRNA (specific to eukaryotes) was sequenced on NextSeq500 System (Illumina; G1, Diel1) or NovaSeq 6000 System (Illumina; G2–3). After quality filtering, paired-end reads were assembled *de novo* with Trinity [38]. Assembled contigs were aligned to MarFERReT v1.1 [37] using DIAMOND v 0.9.18 [39] (-sensitive -b 65 -c 1 -e 1e-5 –top 10 -f100). Taxonomy was assigned by Lowest Common Ancestor (LCA) [39] and the NCBI taxonomic framework. Functional annotation was performed with KofamScan [40], as described in [33]. Contigs supported by ≥10 mapped transcripts were retained. Transcript concentrations were estimated using normalization factors from the synthetic standards [41, 42]. Dinoflagellate transcript concentrations were downscaled by 6.4 [41] to correct for lineage-specific transcript-to-biomass properties.

### Amplicon sequence analysis

Samples for 18S rRNA gene sequencing were collected during G1, 2, and 3. DNA was extracted and processed as described in [32], using DNeasy kits with freeze–thaw, bead-beating, and Proteinase K steps. The V4-V5 region (primers 566F/1200R) was amplified, barcoded, pooled, and sequenced on MiSeq System (Illumina; 250 bp paired-end). Amplicon Sequence Variations (ASVs) were resolved with QIIME2 (v2022.8), and taxonomy was assigned using RESCRIPt with PR2 v5.1. Counts were converted to relative abundance per sample, as published [32]. Only 15 m depth samples were used.

### Flow cytometry biomass and equivalent spherical diameter estimates

Seawater was sampled continuously from the flow-through system at ~7 m depth. Small phytoplankton (<5 μm) were analyzed during G1, 2, and 3 with SeaFlow [30], classified as *Synechococcus*, *Prochlorococcus*, or small eukaryotes based on light scatter and fluorescence, and assigned equivalent spherical diameters (ESDs) using Mie theory [30]. Larger organisms (~3–100 μm ESD) were analyzed during G2 with an Imaging FlowCytobot (IFCb) [31, 41] and classified to phylum level from 2D images. Cell abundance was calculated from particle counts and biovolume; cellular carbon quotas were estimated from ESD using allometric scaling [41]. Carbon (C) biomass was derived from cellular carbon quotas and abundance and averaged over 0.5° latitude. Peak red fluorescence intensity of individual particles was recorded by the IFCB and reported in arbitrary units (a.u.). IFCb-based biomass estimates for 3–5 μm cells may be underestimated due to sensitivity limits [41].

### Statistical tests and visualizations

All analyses were performed in R (v4.5.1, RStudio). Spearman correlations were computed with Hmisc and visualized with ggcorrplot (significance at *P <* .01). ANOVA and ANCOVA (lm, anova) tested regional effects (Gyre, STZ, NTZ) and cruise effects (G1–3). *P* values were corrected for false discovery rate (FDR), and significance terms (FDR < 0.05) were evaluated with *post-hoc* TukeyHSD test. We assessed diel periodicity using Lomb–Scargle [43] and cosinor fitting, considering *P <* .05 significant. Community composition, calculated from the relative abundances of the top 15 ASVs per phylum, was compared across regions using PERMANOVA (adonis2, *vegan*) of Bray–Curtis dissimilarities (999 permutations).

## Results

### Transitions in plankton biomass and size structure across environmental gradients along 158°W

We analyzed the size structure and carbon (C) biomass of the plankton populations across three latitudinal transects in the North Pacific (G1: April 2016; G2: June 2017; G3: April 2019) that transited ~20° along 158°W [31], as well as two high-temporal-resolution studies in the North Pacific Subtropical Gyre (Diel1) [44] and the North Pacific Transition Zone (G3-diel, 2019) [31] (Fig. S1). Sea surface temperature decreased, and inorganic macronutrients increased from south to north along the three cruise transects (Figs 1A for G2, and S2A for G1 and 3). Based on salinity and chlorophyll front locations [31], we distinguished three biomes: (i) the Subtropical Gyre (Gyre), bounded to the north by the 34.82 isohaline, (ii) the Southern Transition Zone (STZ), extending from the Gyre salinity front to the 0.2 mg m^−3^ chlorophyll front, and (iii) the Northern Transition Zone (NTZ), northward of the chlorophyll front (Figs 1A, S2A) [31]. POC and NCP increased by 3- to 10-fold (depending on cruise) north of the chlorophyll front (Figs 1B, S2B) [31]. The observed latitudinal environmental and biogeochemical gradients were all significantly correlated with one another (Spearman, *P <* .01; Fig. S2C).

**Figure 1:**
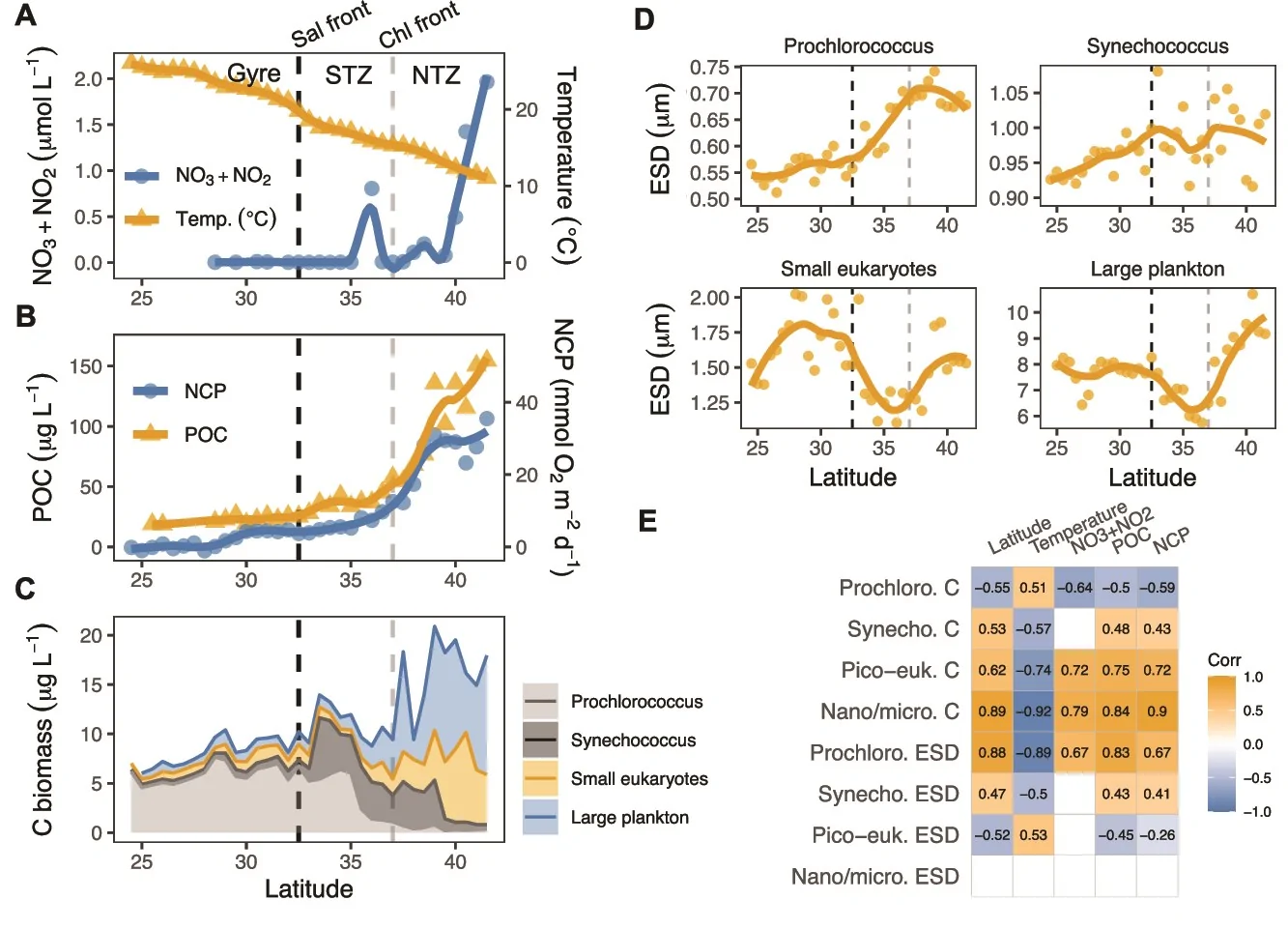
Biogeochemical and plankton structure with latitude (°N) along the Gradients 2 transect. (A) Surface water temperature and inorganic nitrogen concentration (NO_3_ + NO_2_). (B) NCP derived from O₂/Argon ratios, and POC concentrations. (C) Estimated carbon biomass concentrations (mean per 0.5° latitude) of the cyanobacteria *Prochlorococcus*, and *Synechococcus*, and small eukaryotes (0.2–5 μm) from continuous flow cytometry (SeaFlow), and larger plankton (3–100 μm; mostly eukaryotes, blue) from IFCb [31]. (D) Median ESD per 0.5° latitude for *Prochlorococcus*, *Synechococcus*, small eukaryotes (Seaflow), and larger plankton (IFCb). The lines represent a loess smoothing fit of the ESD (span = 0.5). The vertical dashed black lines represent the 34.82 isohaline, marking the boundary between the North Pacific Subtropical Gyre and the STZ. Dashed gray lines indicate the 0.2 mg m^−3^ chlorophyll a threshold, delineating the boundary with the NTZ. (E) Spearman correlations of plankton C biomass and median ESD against environmental variables. Pico-eukaryotic data (Seaflow-derived) are from Gradients 1, 2, and 3; nano/micro-plankton data (IFCb-derived) are from Gradient 2 alone. Correlations with *P <* .01 are shown. The ESD of nano/micro plankton is not significantly correlated with any of the environmental variables.

Abundance and biomass of phytoplankton from 0.5 to 100 μm in equivalent spherical diameter (ESD) were quantified using continuous flow cytometry (SeaFlow) [30] (0.5–5 μm ESD) and imaging flow cytometry (IFCb) [31, 41] (3–100 μm ESD). Seaflow-based biomass estimates varied with latitude across the three transects (Figs 1C, S2D): *Prochlorococcus* cyanobacteria dominated in the oligotrophic Gyre, *Synechococcus* cyanobacteria increased in the STZ as *Prochlorococcus* declined, and small eukaryotes dominated in the nutrient-rich NTZ. Data from IFCb indicate that the biomass of larger plankton also increased in the NTZ. Together, biomass of *Synechococcus*, small eukaryotes (G1–3) and larger plankton (G2) all positively correlated with latitude, POC, and NCP (Spearman, *P <* .01; Fig. 1E), whereas *Prochlorococcus* showed inverse patterns.

Median ESD of the eukaryotic plankton ranged from ~1–2 μm for small photosynthetic eukaryotes (SeaFlow-based) and ~5–11 μm for larger plankton (IFCb-based; Figs 1D, S2E). These eukaryotes encompass a broad range of taxonomically diverse taxa [31]. The lowest eukaryotic ESD values occurred near the chlorophyll front during G2 and G3 (Figs 1D, S2E). ESD of small eukaryotes was negatively correlated with latitude, whereas larger plankton ESD showed no significant correlation (Fig. 1E). The median ESD of *Prochlorococcus* (0.5–0.8 μm) and *Synechococcus* (0.9–1.1 μm) increased with latitude (Fig. 1D and E). Cell size in eukaryotic plankton did not increase consistently along the environmental gradients, as would be expected if nutrient availability directly constrained phototrophic cell size (Fig. 1D and E). These results suggest that latitudinal species turnover and/or shifts in trophic strategies allow larger eukaryotic cells to persist under oligotrophic conditions.

### Transcriptional patterns of dominant eukaryotic plankton along the transect

To determine the balance of different modes of energy acquisition for eukaryotic taxa, we quantified transcript abundances associated with photosynthesis and respiration in poly-A selected metatranscriptomes from small (0.2–3 μm) and large (3–100 μm for G2–3; 3–200 μm for G1) size fractions. Prokaryotic transcripts (e.g. cyanobacteria) were not captured in these datasets. Using the KEGG metabolic pathway framework, we selected a broad set of 564 Kofam annotations from 13 different pathways [40] that represented photosynthesis-related genes required for primary production (photosynthesis, carbon fixation, light harvesting, and chlorophyll and carotenoid biosynthesis) and respiration-related genes required for the tricarboxylic acid (TCA) cycle, oxidative phosphorylation, and the degradation of carbohydrates, lipids, proteins, amino acids, and fatty acids (Table 1, Datasheet S1). Photosynthesis-related transcripts are expected to be restricted to phytoplankton (primary producers), whereas respiration-related transcripts are expected across all organisms, as respiratory substrates may be obtained through phagotrophy, phototrophy, and/or internal recycling. By aggregating across a broad set of genes and pathways, we intend to increase robustness to annotation gaps across taxonomically diverse marine taxa and reduce sensitivity to gene-specific regulatory biases. Only those Kofams with unambiguous pathway assignments were included in the selection.

**Table 1:** Photosynthesis and respiration-related categories based on KEGG classification.

Photosynthesis and carbon fixation	Respiration and catabolism
Carbon fixation in photosynthetic organisms (*n* = 9)	Oxidative phosphorylation (w/o F-type ATPases) (*n* = 132)
Carotenoid biosynthesis (*n* = 30)	TCA cycle (*n* = 45)
Photosynthesis (*n* = 50)	Carbohydrate digestion and absorption (*n* = 90)
Photosynthesis - antenna proteins (*n* = 30)	Fatty acid degradation (*n* = 50)
Porphyrin and chlorophyll metabolism (*n* = 26)	Fat digestion and absorption (*n* = 25)
	Lysine degradation (*n* = 70)
	Protein digestion and absorption (*n* = 53) Valine, leucine, and isoleucine degradation (*n* = 58)

KEGG classifications were further refined by excluding functionally ambiguous KEGG Orthology (Kofam) families. *n* = number of Kofams. The final curated nonredundant list of Kofams included in this study is provided in Datasheet S1.

Twelve taxonomic groups (class/phylum-level) accounted for over 90% of all class/phylum-annotated transcripts associated with the selected photosynthesis and respiration-related Kofams (Datasheet S2). Less than 50% of these reads attained genus-level taxonomic resolution (Datasheet S3), likely reflecting LCA-based assignment to higher taxonomic ranks due to limited representation of open-ocean taxa in reference databases [45, 46]. Consistent with this interpretation, Gradients 1–3 18S rRNA (v4-v5) ASV data [32] revealed substantial diversity within these dominant groups (Datasheet S4). For example, Bacillariophyta and Dinoflagellata were represented by 59 and 176 unique ASVs, respectively, many of which corresponded to genera lacking transcriptomic or genomic references [37]. We therefore focused subsequent analyses mostly at the class/phylum level.

Photosynthesis-related transcripts were detected in seven phototrophic or mixotrophic eukaryotic lineages (Pelagophyceae, Bolidophyceae, Chlorophyta, Dictyochophyceae, Haptophyta, Dinoflagellata, Bacillariophyta), hereafter collectively called “e-phytoplankton” (Figs 2, S3 and S4). Of these, only Bacillariophyta are considered exclusively phototrophic, lacking phagotrophic capacity [17, 26]*.* Photosynthesis-related transcripts were negligible in the 5 heterotrophic lineages (Marine Stramenopiles 3 and 4 (MAST 3-4), Cnidaria, Chordata, Ciliophora, and Arthropoda), collectively called “zooplankton”.

**Figure 2:**
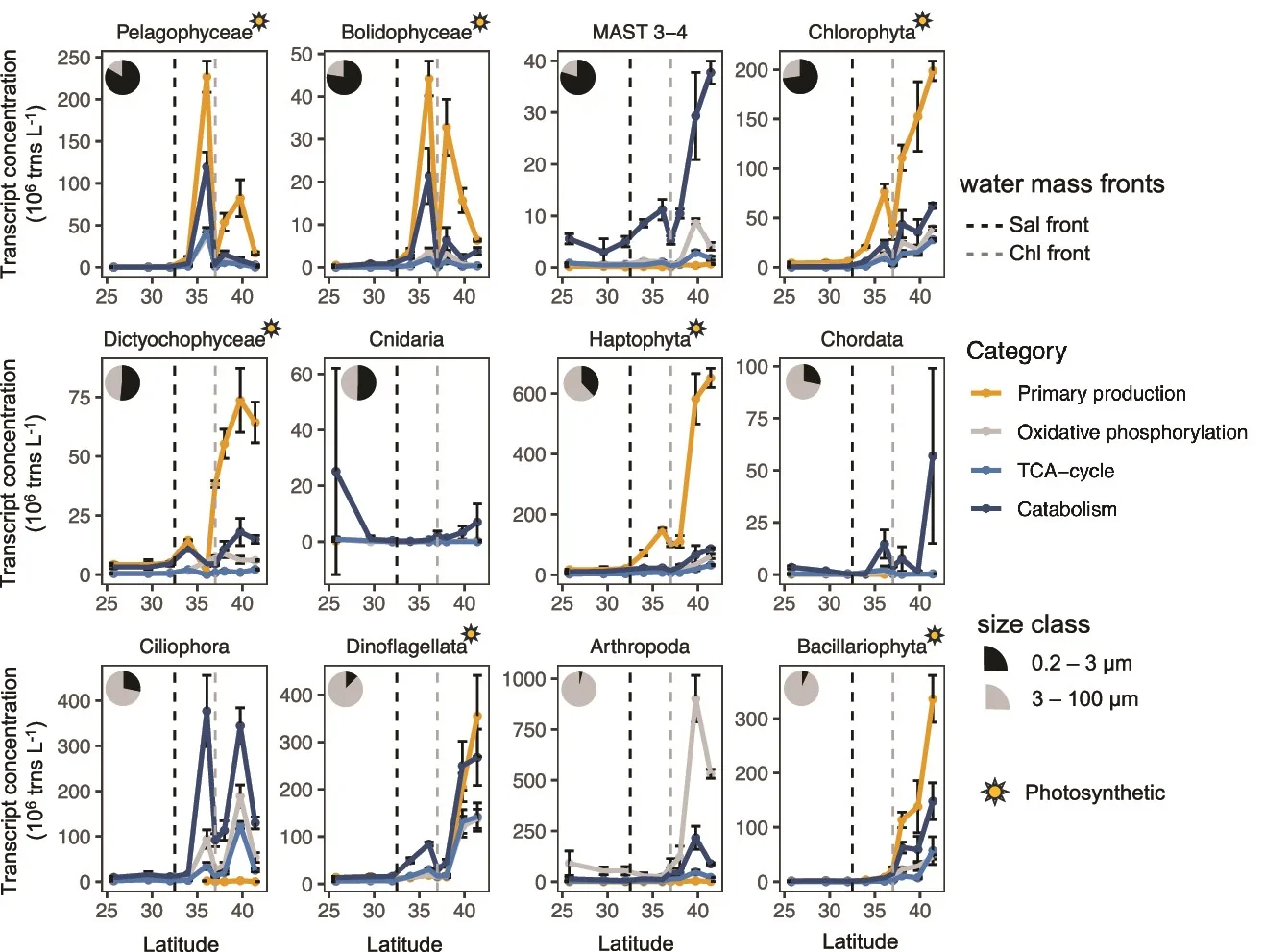
Photosynthesis and respiration transcript abundance for the 12 most abundant eukaryotic plankton taxa along the Gradients 2 transect. Line plots show transcript concentrations (10^6^ transcripts L^−1^) related to primary production, as the sum of photosynthesis, carbon fixation, light harvesting, and chlorophyll and carotenoid biosynthesis (see also Table 1, Datasheet S1), as well as oxidative phosphorylation, TCA cycle, and catabolism. Transcripts from small (0.2–3 μm) and large (3–100 μm) size fractions are combined and displayed as mean ± SD from biological triplicates, with dinoflagellate transcript levels scaled by 1/6.4 [41]. Pie chart insets indicate the mean proportion of transcripts from the small vs. large fraction, and taxa are ordered from predominantly small to large size classes. Sun-symbol indicate photosynthetic taxonomic group. Sampling time was at 04:00 h. Dashed lines denote the salinity and chlorophyll fronts.

Among the e-phytoplankton, Pelagophyceae and Bolidophyceae exhibited peak concentrations of photosynthesis-related transcripts in the STZ that were enriched in the small (<3 μm) size fraction (Figs 2, S3 and S4, inset pie charts). Chlorophyta, Dictyochophyceae, Haptophyta, Dinoflagellata, and Bacillariophyta (ordered from small to large based on relative size fractionations) reached maxima in photosynthesis-related transcript concentrations further north in the NTZ (Figs 2, S3 and S4). Transcript concentrations for respiration-related pathways varied among e-phytoplankton, with Dinoflagellata showing the highest overall levels across all three respiration-related categories (oxidative phosphorylation, TCA cycle, and catabolism).

For zooplankton, respiration-related transcripts were detected in the Gyre for both small protists (MAST 3-4, <3 μm) and large (>3 μm) taxa (Arthropoda, Ciliophora), with transcript concentrations for both groups increasing northward into the TZ (Fig. 2). Arthropoda (multicellular zooplankton) exhibited relatively high oxidative phosphorylation transcript levels compared to unicellular grazers (Fig. 2). Ciliophora transcripts were most concentrated at the chlorophyll front (Fig. 2), whereas Cnidaria and Chordata were detected at low transcript concentrations across the transect.

The estimated transcriptional patterns of photosynthesis and respiration-related genes from the G2 transect across the Gyre, STZ, and NTZ were broadly consistent with those observed during G1 and G3 (Fig. 2 vs. Figs S3 and S4). An analysis of covariance (ANCOVA) across the three cruises showed significant regional differences in the transcript concentrations for all taxonomic groups except Cnidaria (FDR-adjusted *P <* .05; Table S1), with no significant region–cruise interactions except for Dinoflagellata, suggesting that these overall patterns are consistent on an annual basis.

IFCb analysis (G2) confirmed that the major taxonomic groups detected in the large-size metatranscriptomes (Haptophyta, Dinoflagellata, Dictyochophyceae, Bacillariophyta, Ciliophora, and Arthropoda) also dominated biomass, except for the low-abundance Cnidaria and Chordata (Fig. S5). Small e-phytoplankton (<3 μm) were not taxonomically resolved by flow cytometry. Total C biomass of the IFCb-resolved lineages increased with latitude (Fig. S5A), previously found to be proportional to total transcript concentrations [41]. Bacillariophyta median ESD increased from ~9 to 18 μm with latitude (Fig. S5B). Haptophyta and Dinoflagellata median ESD (~5–11 μm) was largest in the Gyre, smallest at the chlorophyll front, and increased in the NTZ (Fig. S5B). Zooplankton were generally larger, with Ciliophora consistently within the nanoplankton size class (<20 μm) and Arthropoda in the microplankton size class (>20 μm). Both e-phytoplankton and zooplankton exhibited increased cellular red-fluorescence (Fig. S5C) and cell size (Fig. S6) north of the chlorophyll front. Red fluorescence may originate from endogenous chloroplasts and/or ingested phytoplankton, precluding discrimination between phototrophic and heterotrophic contributions.

### Northward increase in phototrophy across phytoplankton size classes along the transect

We used the identified photosynthesis and respiration-related transcripts to create the TiP metric as a measure of the transcriptional investment in primary production versus respiration. TiP is defined as the fraction of photosynthesis-related transcripts relative to the total number of photosynthesis and respiration-related transcripts (Table 1, Datasheet S1):

*TiP = (transcripts_photosynthesis_) / (transcripts_photosynthesis + respiration_).*

TiP ranges from 0 to 1, with higher values indicating greater transcriptional investment in primary production and lower values reflecting greater investment in respiration. As such, e-phytoplankton are expected to exhibit higher TiP than zooplankton. Changes in TiP within a given taxon under different environmental pressures are expected to reflect shifts in cellular allocation between photosynthesis and respiration-related pathways. We further expect TiP to be sensitive to organismal and environmental factors, including taxonomy, trophic mode, acclimation to abiotic conditions, and diel regulation of photosynthesis [47].

We examined taxonomic distributions and associated TiP across the Gradients transect sampled at consistent pre-dawn timepoints. The seven dominant e-phytoplankton taxa exhibited distinct latitudinal TiP patterns (Figs 3A, S7A–S8A). Bacillariophyta and Haptophyta TiP was lowest in the Gyre and highest in the NTZ for all cruises (Figs 3B, S7B–S8B). For both taxa, TiP increased directly north of the salinity front in the STZ and continued to increase into the NTZ (Fig. S9). In contrast, Chlorophyta maintained high TiP (>0.6) in the Gyre and STZ, with a slight decline in the NTZ (Fig. S9). Dinoflagellata in G2 had lower TiP (<0.2) within the STZ than within the Gyre or NTZ (>0.3; Fig. 3B), suggesting a shift towards respiration-related processes within the STZ. As expected, TiP of zooplankton taxa remained near zero along the transect. The interannual TiP patterns of Dictyochophyceae and Bolidophyceae were inconsistent (Fig. S9).

**Figure 3:**
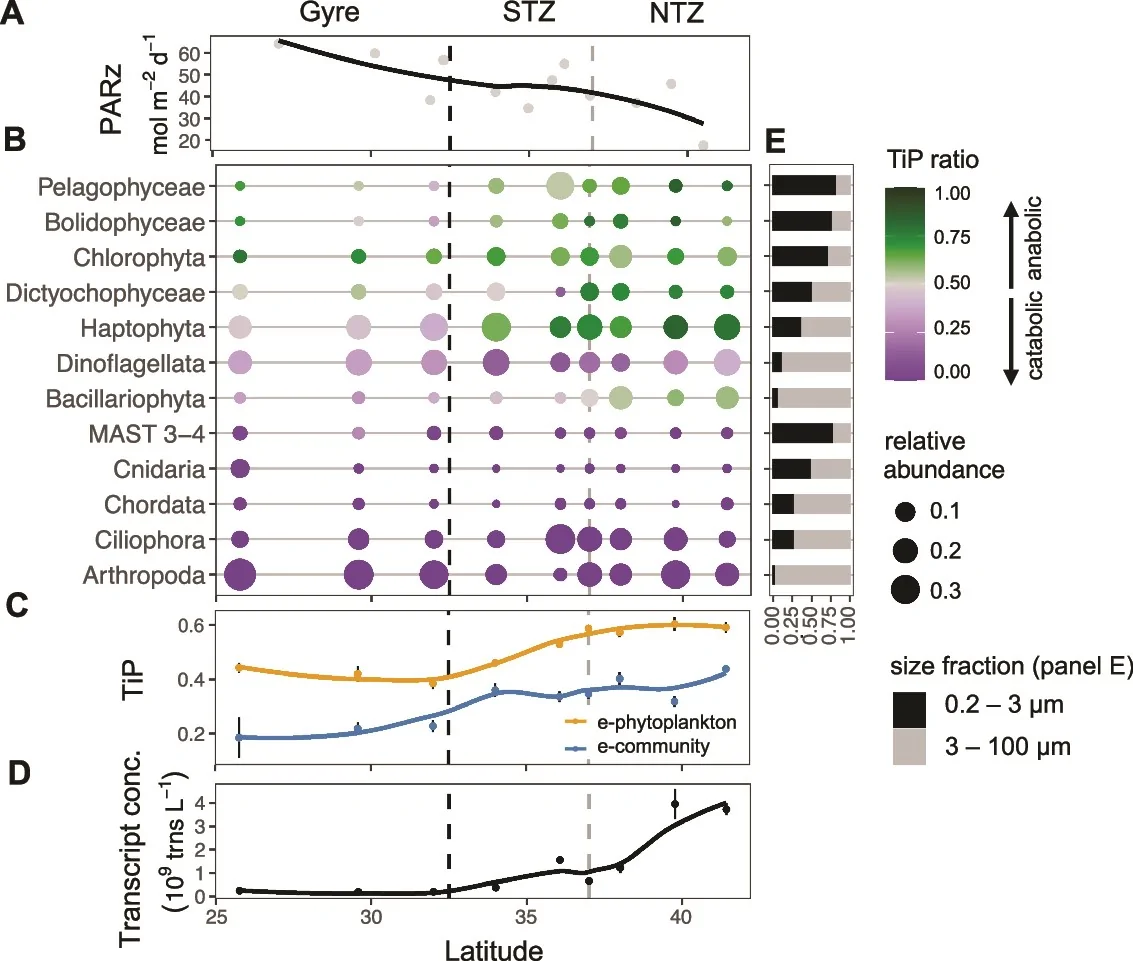
Gradients 2 latitudinal trends in relative transcript abundance and TiP of eukaryotic plankton. (A) Daily-integrated surface photosynthetically active radiation at depth (PARz) estimated per 1 degree latitude. (B) Relative abundance (bubble size) and TiP ratio (color gradient) of eukaryotic plankton. Relative abundance is the fraction of TiP-related transcripts contributed by each taxon among the top 12 taxa. TiP is defined as the fraction of transcripts associated with photosynthesis-related processes specific to primary production (photosynthesis, carbon fixation), relative to the sum of transcripts assigned to both photosynthesis and respiration-related pathways (Table 1, Datasheet S1). Samples collected at 04:00 h. Dinoflagellate values were scaled by a factor of 1/6.4 based on transcript-to-biomass estimates [41]. (C) Community-level (combined top 12 taxa) and phytoplankton-level (combined photosynthesizing phyla) TiP, with error bars representing the standard deviation. (D) Estimated transcript concentrations (TiP-assigned transcripts only) of the combined top12 taxa. Dashed lines denote the salinity and chlorophyll front. (E) Transcript distributions across small (0.2–3 μm) and large (3–100 μm) filter size fractions.

Total e-phytoplankton and total “e-community”-level TiP were calculated by summing TiP transcripts across e-phytoplankton or from the combined e-phytoplankton and zooplankton. The e-phytoplankton TiP increased by about 1.5-fold, from ~0.4–0.5 in the Gyre to ~0.6–0.7 in the NTZ across all cruises (Figs 3C, S7C–S8C). e-Community TiP also increased northward, consistent with coupled changes in the dominant e-phytoplankton and zooplankton. These TiP increases were accompanied by increases in total transcript concentrations (Figs 3D, S7D–S8D).

We evaluated whether the latitudinal TiP patterns were constrained by cell size as classified by filter size-fractionation. Pelagophyceae, Bolidophyceae, and Chlorophyta were mostly recovered in the small size fraction (<3 μm; Figs 3E, S7E–S8E). Within these taxonomic groups, the dominant small-sized genera were *Pelagomonas* (Pelagophyceae), *Triparma* (Bolidophyceae), and *Micromonas* and *Bathycoccus* (Chlorophyta) (Figs S10–S12). Haptophyta and Dictyochophyceae transcripts were found across both small and large (>3 μm) size fractions and included *Phaeocystis* and *Chrysochromulina* (Haptophyta) and *Dictyocha* (Dictyochophyceae). The Dinoflagellate and Bacillariophyta transcripts were primarily collected in the large fraction (Figs 3E, S7E–S8E) and were more diverse than the other taxa such that no individual annotated genus represented more than 5% of the sequence reads. For a given taxon, there was no relation between their cell size fractionation and the latitude at which TiP first increased. Instead, we found that the small e-phytoplankton (e.g. Chlorophyta) generally exhibited higher TiP in the Gyre than the larger phytoplankton, and lower TiP in the NTZ (Figs 3B, S7–S9). Zooplankton-derived TiP transcripts were detected in both the small (MAST 3-4) and large size fractions (Chordata, Ciliophora, and Arthropoda), with Cnidaria (*Hydra*) captured equally in each fraction (Figs 3, S7 and S8).

### Diel periodicity of TiP in Gyre and TZ plankton communities

We examined TiP over the day-night cycle to quantify variation associated with light-driven and circadian regulation of photosynthetic investment [45, 47]. TiP was measured over diel cycles at two sites at the extremities of the Gradients transect: the North Pacific Subtropical Gyre at 25.4° N (Diel1, July 2015) [45], and the North Pacific Transition Zone at 41.6° N (G3-diel, April 2019) [33]. Multi-day semi-Lagrangian sampling enabled assessment of TiP reproducibility across the day-night cycle. The Gyre had warm, nutrient-depleted surface waters (26.8°C; <0.02 μmol L^−1^ NO₃ + NO₂); the NTZ was colder and more macronutrient-replete (9.8°C; 6.2 μmol L^−1^ NO₃ + NO₂). Photosynthetically active radiation at sampling depth (PARz) reached ~1000 μmol photons m^−2^ s^−1^ in the Gyre and ~750 μmol photons m^−2^ s^−1^ in the NTZ at local noon (Fig. 4A and B).

**Figure 4:**
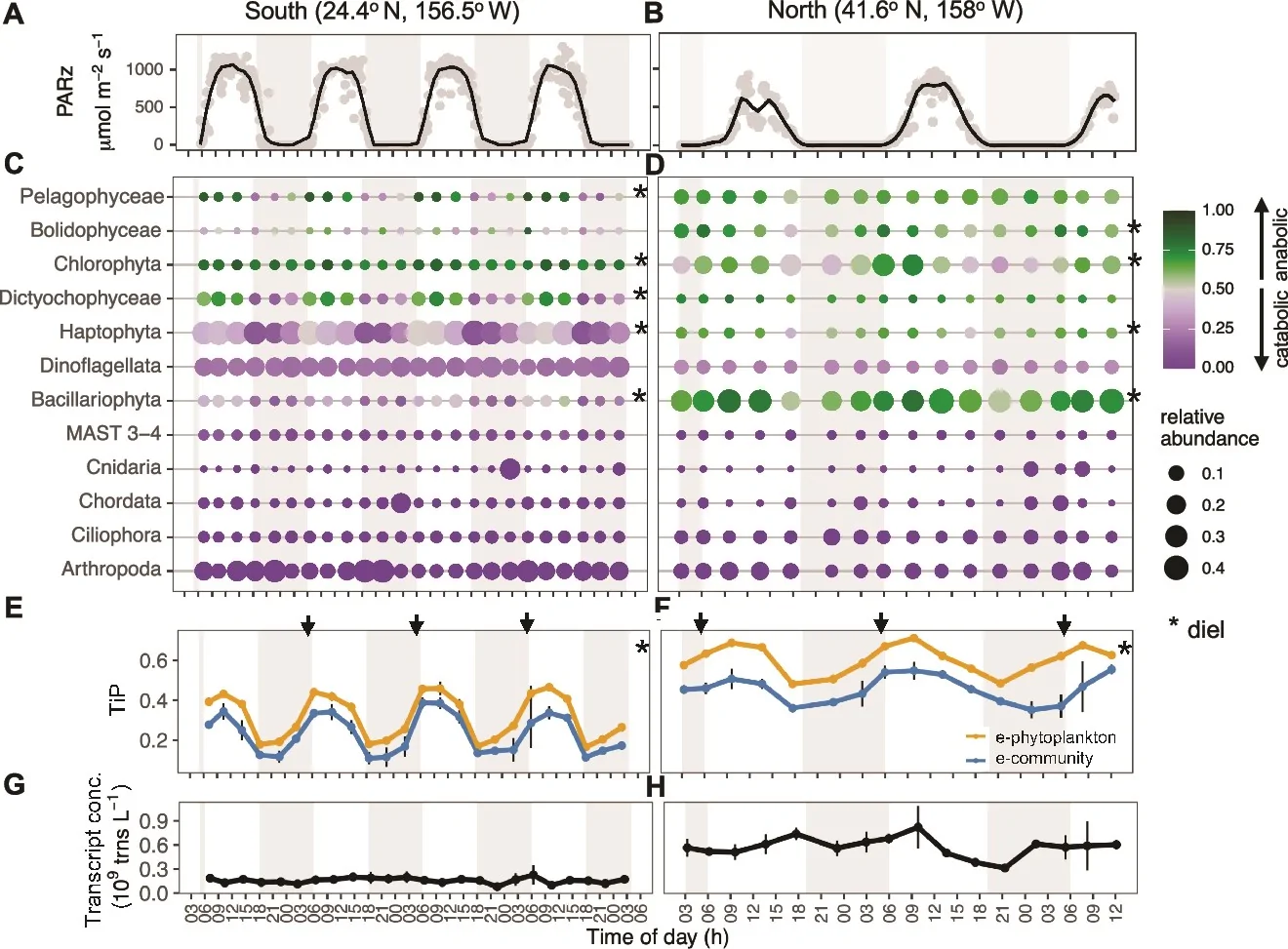
Diel fluctuations in relative abundance and TiP ratio of Gyre and NTZ eukaryotic plankton. (A, B) Estimated photosynthetically active radiation at 15 m depth (PARz) during the Diel1 cruise (July 2015, 24.4°N, 156°W) and G3-diel cruise (April 2019, 41.6°N, 158°W). Datapoints indicate mean PARz, calculated for 15 m depth. (C, D) Relative TiP-assigned transcript abundance (bubble size) and TiP ratio (bubble color) of the dominant 12 taxonomies for Diel1 and G3-diel. Relative abundance is the fraction of TiP-related transcripts contributed by each taxon among the top 12 taxa. Dinoflagellate values were scaled by a factor of 1/6.4 based on transcript-to-biomass estimates [41]. (E, F) Community-level (combined top 12 taxa) and phytoplankton (combined photosynthesizing phyla) TiP for Diel1 and G3-diel, with error bars representing the standard deviation. (G, H) Estimated transcript concentrations of the combined top12 taxa. Samples were collected approximately every 4 h during Lagrangian drifter deployments. Shaded regions indicate night periods; arrows mark dawn sampling times corresponding to Gradients transects. Asterisks (*) to the right of each panel denote trends with significant diel periodicity as determined by Lomb–Scargle analysis (*P <* .05; see Table 2) [43].

e-Phytoplankton TiP varied predictably over the diel cycle, with higher TiP during the daylight hours and lower values during the night (Fig. 4), consistent with diel modulation of photosynthesis [47]. Lomb–Scargle analysis [43] confirmed significant diel periodicity for most e-phytoplankton at one or both locations, except for Dinoflagellata (Table 2, Figs S13 and S14), consistent with reduced transcriptional and increased post-transcriptional regulation by this phylum [48]. TiP values peaked between 9 and 11 h in the Gyre and between 6 and 10 h in the NTZ (Table 2). At both diel sites, estimated peak times were later for Bacillariophyta and Chlorophyta relative to Haptophyta (Table 2). This shift was consistent between sites and may reflect differences in light sensing and circadian regulation [49], or divergent physiological acclimation to diel fluctuations. Overall, the diel variation (amplitude) in e-phytoplankton TiP was greater in the Gyre than in the NTZ, driven by lower nighttime values. TiP for zooplankton remained near zero throughout the day at both locations without a diel signature (Fig. 4C and D, Table 2).

**Table 2:** TiP diel periodicity parameters for the 12 dominant taxa during the Diel1 (South) and G3-diel (North) cruises.

Taxon	Diel1 TiP (amp)	Diel1 period (h)	Diel1 peak time (h:m)	G3-diel TiP (amp)	G3-diel period (h)	G3-diel peak time (h:m)
Pelagophyceae	0.58 (0.36)	24.0	09:10	0.64	–	–
Bolidophyceae	0.29	–	–	0.63 (0.13)	23.7	06:23
Chlorophyta	0.79 (0.04)	23.4	10:53	0.52 (0.15)	23.7	09.27
Dictyochophyceae	0.44 (0.31)	24.0	10:07	0.69	–	–
Haptophyta	0.30 (0.19)	24.7	09:06	0.59 (0.09)	23.7	07:14
Dinoflagellata	0.20	–	–	0.26	–	–
Bacillariophyta	0.30 (0.17)	24.0	10:59	0.67 (0.10)	23.7	09:28
MAST 3-4	0.07	–	–	0.01	–	–
Cnidaria	0.00	–	–	0.00	–	–
Chordata	0.00	–	–	0.00	–	–
Ciliophora	0.00	–	–	0.00	–	–
Arthropoda	0.00	–	–	0.00	–	–
Phototrophs	0.32 (0.15)	24.0	09:29	0.59 (0.10)	23.7	09:13
Community	0.23 (0.13)	24.7	09:44	*0.45 (0.08)*	*27.1*	*10:46*

Periodicity was assessed using Lomb–Scargle periodograms [43] and cosinor fitting, with significance defined as *P* < .05. Average TiP (Midline Estimating Statistic Of Rhythm; MESOR) is given with amplitude in parentheses for those taxa with significant periodicity. Period length and peak time of the TiP metric are also provided. Values in italics indicate a periodicity outside the range of 23–25 h.

Average TiP was higher for e-phytoplankton than zooplankton and varied by taxon, location, and time (Fig. 4C and D, Table 2). Within the Gyre during daylight hours, Pelagophyceae, Chlorophyta, Dictyochophyceae, and Bolidophyceae exhibited maximum TiP exceeding 0.5 (Fig. S13). The highest TiP was observed for Pelagophyceae and Chlorophyta, which exceeded 0.8. Haptophyta, Bacillariophyta, and Dinoflagellata exhibited daytime TiP below 0.5 in the Gyre, suggesting greater transcriptional investment in respiration relative to photosynthesis under low-nutrient, high-light conditions. This pattern may reflect photosynthetic acclimation or, in mixotrophic taxa, a shift toward greater heterotrophic resource acquisition. Dinoflagellata maintained a low TiP (~0.2) throughout the diel cycle (Fig. 4C and D, Table 2), as did the abundant Haptophyta (0.30 ± 0.19). TiP for most phytoplankton were significantly higher at the NTZ station than in the Gyre. Bacillariophyta TiP in the NTZ was 0.67 ± 0.10, compared to 0.30 ± 0.17 in the Gyre. Haptophyta averaged 0.59 ± 0.09 and Dinoflagellata TiP increased marginally to ~0.29 (Table 2). ANOVA indicated that differences in TiP between Gyre and NTZ datasets were significant for all phytoplankton except Pelagophyceae (FDR-adjusted *P <* .05; Table S2).

Total e-phytoplankton and e-community TiP were higher in the NTZ than in the Gyre, a result of the accumulative effects of shifts in community composition and TiP (Figs 4E and F, S13 and S14). Haptophyta and Dinoflagellata with low TiP dominated eukaryotic plankton transcripts in the Gyre, and Bacillariophyta and Chlorophyta with high TiP dominated in the NTZ. At both sites, e-phytoplankton TiP exhibited pronounced diel periodicity, a pattern that was also evident at the community level. The offset between e-phytoplankton and e-community-wide TiP was 0.09 ± 0.03 in the Gyre and 0.15 ± 0.05 in the NTZ, indicating higher contribution of e-phytoplankton to e-community-wide TiP in the NTZ. Community-level transcript concentrations were also higher in the NTZ (Diel1: 0.16 ± 0.05 × 10^9^; G3-diel: 0.59 ± 0.16 × 10^9^ transcripts L^−1^; Fig. 4G and H) but lacked significant diel periodicity.

### Correlations of Transcript-informed Productivity to environmental drivers and transcript abundance

To assess relationships between TiP and environmental parameters, we performed Spearman correlations on the combined Gradients dataset, supplemented with dawn samples from the two diel studies (*n* = 35). Bacillariophyta TiP was positively correlated with latitude, inorganic nitrogen, POC, and NCP, and negatively correlated with temperature and PARz (*P <* .01; Fig. 5A). Haptophyta TiP showed positive correlations with latitude, POC, and NCP. TiP for other taxa exhibited no significant relationships with these variables. However, the combined e-phytoplankton TiP was significantly correlated with all environmental parameters examined (Fig. 5A). POC and NCP integrate whole-community processes, including prokaryotic contributions, whereas TiP reflect eukaryotic productivity alone. Total e-community TiP showed no consistent latitudinal trend but was significantly correlated with inorganic nitrogen and POC (Fig. 5A). Because environmental variables were strongly co-correlated along the transect (Fig. S2C), individual drivers cannot be resolved.

**Figure 5:**
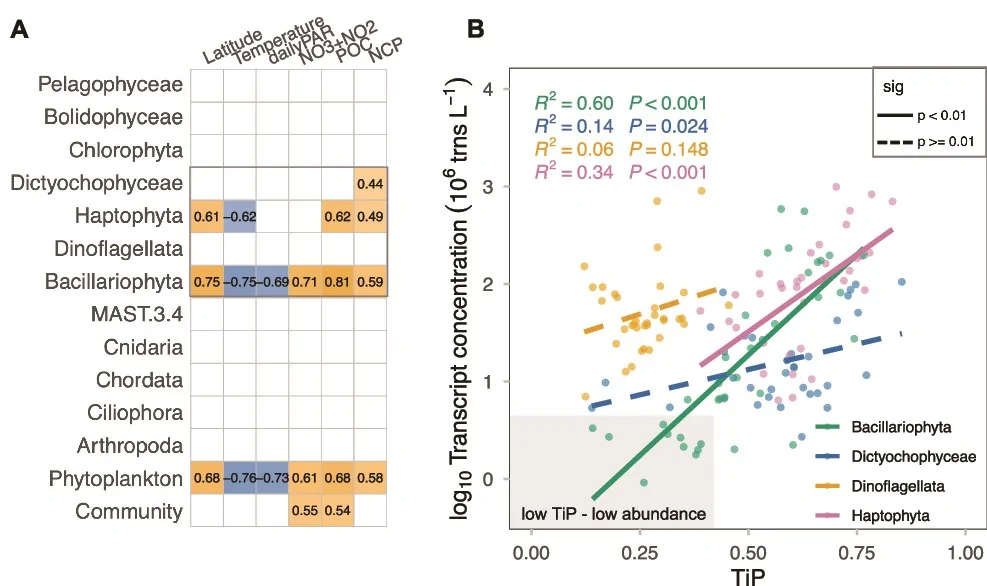
Correlations of TiP to environmental features and transcript abundance. (A) Spearman’s rank correlation coefficients (rs) between metatranscriptome-derived TiP and environmental variables: latitude, temperature, daily-integrated PAR(z), inorganic nitrogen concentration (NO₃ + NO₂), POC, and NCP for the top 12 taxa and the combined e-phytoplankton and total e-community. Only correlations with *P <* .01 are shown. (B) Scatterplot of log₁₀-transformed taxon-level transcript concentrations (TiP-assigned transcripts only) versus TiP. Linear regression lines are shown, with corresponding regression equations, R^2^ values, and *P* values. Solid lines indicate significant relationships (*P <* .01), dashed lines indicate nonsignificant relationships (*P* ≥ .01). The dataset comprised 35 metatranscriptome sampling stations (mean of biological replicates) from Gradients 1 ~ 3 and Diel1 and G3-diel AM samples (*n* = 35).

We examined the relationship between TiP values and TiP-related transcript abundance in the four nano-sized e-phytoplankton taxa: Dictyochophyceae, Haptophyta, Dinoflagellata, and Bacillariophyta (Fig. 5B). We reasoned that higher transcript abundance would reflect growth under more favorable conditions [12, 50]. Accordingly, higher TiP is expected to coincide with higher transcript abundance for obligate phototrophs, whereas this relationship may be weaker in mixotrophic taxa that supplement photosynthesis through grazing. Transcript concentrations of Bacillariophyta and Haptophyta were positively correlated with TiP (*P* < .01; Fig. 5B). Further, despite their obligate phototrophy, Bacillariophyta exhibited the widest range of TiP and transcript concentrations, with high values in the NTZ, but low TiP (<0.4) associated with low transcript abundance (Fig. 5B, gray-shaded box) and low biomass (Fig. S5A) in the Gyre, suggestive of unfavorable growth conditions such as high light exposure and nutrient limitation. No significant relationship was observed for Dinoflagellata or Dictyochophyceae. Dinoflagellata maintained low TiP despite relatively high transcript concentrations, consistent with mixotrophic or heterotrophic nutritional strategies [17]. Haptophyta did not exhibit the low TiP values observed in Bacillariophyta and Dinoflagellata, further highlighting taxon-specific relationships between TiP and transcript abundance.

### Amplicon-based resolution of taxonomic successions and latitudinal biogeography

The observed latitudinal patterns in TiP, cellular abundance, and median ESD within taxonomic groups may reflect species succession, physiological plasticity of ubiquitous strains, or both. Because the metatranscriptome taxonomic resolution is limited to taxa that have sequenced genomes or transcriptomes, we leveraged size-fractionated (0.2–3 μm and >3 μm) 18S rRNA (v4-v5) ASV data available for G1, G2, and G3 [32] mapped against the PR2 database [51] to determine how the patterns of the dominant large- and small-sized e-phytoplankton varied across the transects.

Within the large-size fraction, the most abundant Haptophyta ASVs were assigned to *Chrysochromulina* (six distinct ASVs in G2) and *Phaeocystis* (three distinct ASVs in G2; Figs 6A, S15 and S16, Datasheet S5), consistent with the metatranscriptomes (Figs S10–S12). Two ASVs, assigned to *Chrysochromulina* and Noelaerhabdaceae, were ubiquitous along the transect, whereas most ASVs were regionally restricted (Fig. 6A, indicated by asterisks). Also identified were Braarudosphaeraceae X and HAP3 ASVs, two lineages absent from our transcriptome references [37] and therefore not recognized at genus level in the metatranscriptomes. Most dinoflagellate ASVs also belonged to genera that are not recognized in the metatranscriptomes, namely *Gyrodinium*, *Warnowia*, *Biecheleria*, as well as members of Syndiniales groups I and II (Figs 6A, S15 and S16). Of these, *Gyrodinium* and *Warnowia* ASVs were ubiquitous along the transect, whereas others, such as *Biecheleria*, displayed more restricted biogeography. Contrary to Haptophyta and Dinoflagellata, the Bacillariophyta ASVs were almost all located north of the salinity front into the NTZ and included pennate (e.g. *Cylindrotheca, Fragilariopsis*, and *Pseudo-nitzschia*) and centric diatoms (all others; Fig. 6A).

**Figure 6:**
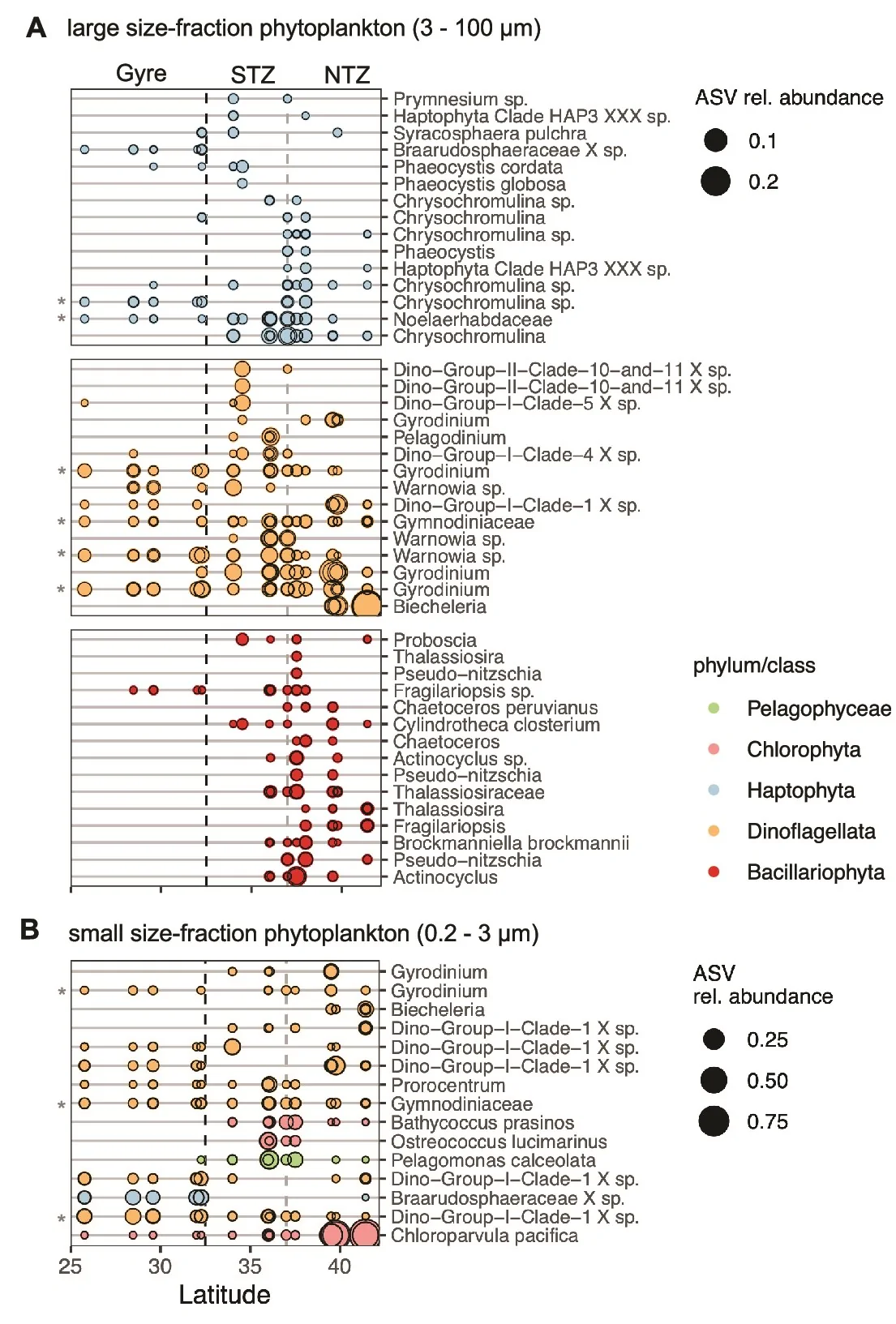
ASV-level taxonomic succession of eukaryotic phytoplankton along Gradients 2. (A) Large size fraction (3–100 μm): Haptophyta (top), Dinoflagellata (middle), and Bacillariophyta (bottom), showing the 15 most abundant and unique ASVs per taxonomic group. (B) Small size fraction (0.2–3 μm): top 15 ASVs of small primary producers. Bubble plots represent ASVs based on the 18S rRNA V4-V5 region, with bubble size proportional to relative abundance at each site, calculated within each size fraction [32]. Taxonomies were assigned using PR2 v5.1 at the most precise rank supported by the sequence [32]. ASVs were ordered using hierarchical clustering (Ward’s method; ward.D). ASV feature IDs and taxon strings are given in Datasheet S5. Asterisks on the left side indicate ubiquitous ASVs with consistent relative abundance across the transect.

Within the small-size fraction, a single ASV assigned to *Braarudosphaeraceae* X sp. (Haptophyta) dominated in the Gyre (Figs 6B, S15 and S16). Other Gyre-associated ASVs were of dinoflagellate origin and exhibited more ubiquitous distributions along the transect. Additional small-size fraction ASVs included the small-sized e-phytoplankton *Pelagomonas calceolata* (Pelagophyceae), and *Ostreococcus marinus* and *Bathycoccus prasinos* (Chlorophyta), which were concentrated near the chlorophyll front and coincided with the smallest ESD as captured by Seaflow (Fig. 1D). *Micromonas* was identified among the top 15 ASVs in G1 and G3, but not G2. In the northernmost region, *Chloroparvula pacifica* dominated the NTZ in G2–3, but not G1 (Figs 6B, S16B). PERMANOVA analysis on relative ASV abundance data indicated that 17%–24% of variation in top 15 primary producer composition was explained by region (Gyre, STZ, NTZ; *P <* .01, Table S3), with the remainder occurring within regions. These results suggest that latitudinal patterns in community structure and function are associated with regional species turnover, whereas local-scale variability among sampling sites also contribute substantially.

## Discussion

### Transcript-inferred Productivity metric and primary production

We developed the TiP metric to improve mechanistic and taxonomic insight into ecosystem productivity, not commonly resolved by bulk measurements such as NCP [52] or satellite products [53]. TiP quantifies taxon-specific nuclear-encoded transcriptional allocation to photosynthetic and respiratory pathways of eukaryotic plankton. Analyses of metatranscriptomes across environmental gradients and diel cycles indicate that (i) eukaryotic phytoplankton TiP exhibits consistent diel patterns, demonstrating that the metric is reproducible when sampled at consistent times of day, and (ii) eukaryotic community-level TiP responds predictably to environmental pressures: warmer oligotrophic waters are dominated by organisms with lower TiP (more reliance on respiration), and cooler nutrient-rich waters are dominated by organisms with higher TiP (more reliance on photosynthesis) [54]. The diel patterns in e-phytoplankton TiP reflect the day-night regulation of photosynthesis [47] and are consistent with previous observations of diel periodicity in oxidative phosphorylation, carbon fixation, and photosynthesis-related gene expression in marine phytoplankton [45, 55].

The differences between eukaryotic phytoplankton (e-phytoplankton) TiP and total eukaryotic community TiP (including zooplankton) was larger in the NTZ than in the Gyre during diel studies (Fig. 4), indicating that e-phytoplankton productivity outpaced zooplankton consumption in the NTZ [56]. This pattern is consistent with the net accumulation of e-phytoplankton biomass (Fig. 1C) and may reflect transient decoupling of phytoplankton growth and grazing characteristic of productive oceanic systems [57]. The smaller TiP difference in the Gyre suggests tighter coupling between production and consumption, consistent with stable POC concentrations (Fig. 1B) and previous observations of tightly coupled diel metabolic processes among microbial community members at this location [53]. The positive correlation between TiP and bulk NCP in the NTZ (Fig. 5) suggests that TiP can capture broad trends in metabolic activity. Because NCP integrates whole-community productivity and respiration, whereas TiP reflects only the eukaryotic component, this relationship should be interpreted cautiously and requires validation against taxon-specific carbon fixation and respiration rates.

### Taxon-specific interpretation of Transcript-informed Productivity

Although community TiP increased northward with nutrient availability, different taxa contributed unevenly to this pattern. The highest TiP values were observed for Chlorophyta, Pelagophyceae, Dictyochophyceae, Haptophyta, and Bacillariophyta, groups composed primarily of phototrophic and/or mixotrophic taxa, supporting their role in primary productivity. However, TiP values for these taxa, except Chlorophyta, varied markedly both across taxa and along environmental conditions.

Bacillariophyta showed a particularly broad range in TiP along the transect despite being composed almost exclusively of photosynthetic species [58]. The low TiP values of Gyre Bacillariophyta, where they occur at low abundance, may reflect photosynthetic acclimation to low nutrient conditions. This interpretation is consistent with diatom culture studies showing that nutrient stress reduces PSII photochemical efficiency (Fv/Fm) and O₂ production and increases respiration [59, 60], collectively yielding a physiology expected to result in low TiP. These observations suggest that low TiP can reflect the physiological state of obligate phototrophs and therefore requires interpretation within the context of trophic capacity and ecological state. Low TiP combined with low cellular abundance may indicate prolonged nutrient stress leading to a dormant or autophagy-associated state [61], a hypothesis that could be evaluated in field populations by examining autophagy-related gene expression patterns in future studies [27].

We hypothesize that low TiP combined with high transcript abundance in mixotrophic organisms reflects metabolism supported by resources acquired through phagocytosis. For example, Dinoflagellata displayed consistently low TiP values (<0.3). These “middle-class” nanoplankton (ESD < 10 μm), with predominantly mixotrophic and/or heterotrophic capabilities [17, 19], dominated the particle size distribution of this region and are considered ecologically significant [31]; yet, their exact contributions to carbon production and biogeochemical cycling remain uncertain. Low TiP values observed for Dinoflagellata suggest a primarily consumer role, with limited contribution to net primary productivity but likely enhancing transfer to higher trophic levels [8]. This interpretation is supported by ASV-based trophic assignments, which identify several abundant dinoflagellate lineages, including *Gyrodinium* and Group-I dinoflagellates, as heterotrophic or parasitic [18].

Haptophyta, containing both mixotrophic and phototrophic members, were ubiquitous along the transect, but exhibited low TiP in the Gyre and elevated TiP north of the salinity front. This suggests a northward shift along the trophic continuum toward phototrophy, potentially driven by species turnover and/or physiological plasticity. The transect contained several ubiquitous *Chrysochromulina* ASVs (Fig. 6) that persisted across the entire transect, some of which also recurred interannually [32]. *Chrysochromulina* isolates from this region were shown to ingest *Prochlorococcus* and other bacteria [15, 62] and were predicted by machine-learning to adjust trophic strategy over environmental gradients [23], indicative of ecological versatility. Other Haptophyte species showed more restricted latitudinal distributions. ASVs affiliated with Braarudosphaeraceae were dominant but confined to the Gyre (Figs 6, S15 and S16). This lineage includes *Braarudosphaera bigelowii*, which can fix N₂ via a UCYN-A-derived nitroplast [63, 64], an alternate trait that may facilitate persistence under oligotrophic conditions.

Phototrophic specialists were generally confined to higher-nutrient regions compared to mixotrophic taxa. Examples include pico-eukaryotic *Pelagomonas*, *Ostreococcus*, and *Bathycoccus* in the STZ at the Chlorophyll front, and *Chloroparvula* (Chlorophyta), *Fragilariopsis* and *Thalassiosira* (Bacillariophyta), and *Biecheleriopsis* (Dinoflagellata) in the NTZ (Fig. 6). These taxa are primarily obligate phototrophs associated with higher-nutrient regimes [32, 65].

Together, three TiP-related patterns emerged: (i) high TiP and high transcript abundance, indicative of active phototrophic production; (ii) low TiP and low transcript abundance, consistent with reduced activity or carbon recycling; and (iii) low TiP but high transcript abundance, suggestive of mixotrophic or heterotrophic strategies that decouple carbon acquisition from photosynthetic investment. Thus, whereas TiP alone does not categorically resolve organismal trophic mode, insight into metabolic strategies and relative contributions to community productivity can be gained by integrating TiP with abundance patterns and prior knowledge of organismal trophic capacity.

### Toward explicit representation of mixotrophy in ecosystem models

Phytoplankton population size structure is classically interpreted as the outcome of bottom-up constraints, such as nutrient availability and temperature [10–13], modulated by biotic interactions, including competition for limiting resources, as well as top-down constraints, such as selective grazing by zooplankton [66, 67]. These processes influence both phytoplankton biomass and cell-size distributions across environmental gradients. In this framework, small phototrophic cells, with high surface-to-volume ratios and low subsistence nutrient requirements, are favored in oligotrophic environments, whereas larger phototrophic cells are associated with higher nutrient availability and top-down control on the smaller cells [10–13]. Along our transect, cell-size succession from *Prochlorococcus* in the Gyre, to *Synechococcus* and pico-eukaryotes (Chlorophyta, Pelagophyceae) at the chlorophyll front, and Bacillariophyta in the northernmost stations is consistent with the coupled bottom-up/top-down size paradigm. However, our results also suggest that mixotrophy introduces additional axes of control, with nano-sized mixotrophs remaining abundant across the nutrient gradient. By acquiring nutrients through prey ingestion [8, 9], mixotrophs may sustain larger cell sizes, biomass and transcriptional activity under oligotrophic conditions where nutrient limitation constrains phototrophic growth.

By decoupling cell size and biomass from photosynthetic performance while simultaneously imposing enhanced respiratory and catabolic demands associated with grazing [8, 9], mixotrophy complicates traditional ecological frameworks. The TiP metric may provide a quantitative entry point for developing dynamic size-productivity scaling relationships that explicitly incorporate metabolic plasticity. Such relationships can be tested under controlled laboratory conditions. Explicit representation of mixotrophic nanoplankton in ecosystem models will be important for accurately resolving net community production and carbon flow through the microbial food web.

## Supplementary Material

Supplementary_material_wrag234

## Data Availability

The code used for quantitative metatranscriptomics is available at https://github.com/armbrustlab/NPac_euk_gene_catalog. The metatranscriptome transcript counts, taxonomic and functional annotations are available at https://zenodo.org/records/13826820 and https://zenodo.org/records/12630398. IFCb-derived datasets can be found at https://zenodo.org/records/4267135 and https://zenodo.org/records/4267140. SeaFlow v1.6 datasets are archived on Zenodo (https://doi.org/10.5281/zenodo.10896099). ASV Data are available at https://zenodo.org/records/17288024. Additional metadata and associated data sets are available at the Simons CMAP ocean data portal [34] here: SCOPE Diel1 associated data: https://simonscmap.com/catalog/cruises/KM1513. Gradients 1 associated data: https://simonscmap.com/catalog/cruises/KOK1606. Gradients 2 associated data: https://simonscmap.com/catalog/cruises/MGL1704. Gradients 3 and G3-diel associated data: https://simonscmap.com/catalog/cruises/KM1906.

## References

[1] Field CB, Behrenfeld MJ, Randerson JT, et al. Primary production of the biosphere: integrating terrestrial and oceanic components. *Science* 1998;281:237–40. 10.1126/science.281.5374.237 9657713

[2] Keeling PJ . Diversity and evolutionary history of plastids and their hosts. *Am J Bot* 2004;91:1481–93. 10.3732/ajb.91.10.1481 21652304

[3] Delwiche CF . Tracing the thread of plastid diversity through the tapestry of life. *Am Nat* 1999;154:S164–77. 10.1086/303291 10527925

[4] Mitra A, Flynn KJ, Burkholder JM, et al. The role of mixotrophic protists in the biological carbon pump. *Biogeosciences* 2014;11:995–1005. 10.5194/bg-11-995-2014

[5] Mitra A, Flynn KJ, Tillmann U, et al. Defining planktonic protist functional groups on mechanisms for energy and nutrient acquisition: incorporation of diverse mixotrophic strategies. *Protist* 2019;167:106–20. 10.1016/j.protis.2016.01.003 26927496

[6] Guo Z, Zhang H, Liu S, et al. Biology of the marine heterotrophic dinoflagellate Oxyrrhis marina: current status and future directions. *Microorganisms* 2013;1:33–57. 10.3390/microorganisms1010033 PMC5029500 27694763

[7] Worden AZ, Follows MJ, Giovannoni SJ, et al. Rethinking the marine carbon cycle: factoring in the multifarious lifestyles of microbes. *Science* 2015;347:347. 10.1126/science.1257594 25678667

[8] Ward BA, Follows MJ. Marine mixotrophy increases trophic transfer efficiency, mean organism size, and vertical carbon flux. *Proc Natl Acad Sci US A* 2016;113:2958–63. 10.1073/pnas.1517118113 PMC4801304 26831076

[9] Chakraborty S, Nielsen LT, Andersen KH. Trophic strategies of unicellular plankton. *Am Nat* 2017;189:E77–90. 10.1086/690764. 28350501

[10] Marañón E . Cell size as a key determinant of phytoplankton metabolism and community structure. *Annu Rev Mar Sci* 2015;7:241–64. 10.1146/annurev-marine-010814-015955 25062405

[11] Chisholm SW . Phytoplankton size. In: Falkowski PG, Woodhead AD, Vivirito K, eds. Primary Productivity and Biogeochemical Cycles in the Sea. Boston, MA: Springer US, 1992, 213–37 10.1007/978-1-4899-0762-2_12.

[12] Finkel ZV, Beardall J, Flynn KJ, et al. Phytoplankton in a changing world: cell size and elemental stoichiometry. *J Plankton Res* 2010;32:119–37. 10.1093/plankt/fbp098

[13] Hilligsøe KM, Richardson K, Bendtsen J, et al. Linking phytoplankton community size composition with temperature, plankton food web structure and sea–air CO2 flux. *Deep-Sea Res I Oceanogr Res Pap* 2011;58:826–38. 10.1016/j.dsr.2011.06.004

[14] Rihm G, Benedetti F, Bittner L. Do trophic strategies shape biogeography and environmental niches? Marine dinoflagellates as a case study. *ISME Commun* 2025;5:5. 10.1093/ismeco/ycaf153 PMC12452278 40989910

[15] Li Q, Edwards KF, Schvarcz CR, et al. Broad phylogenetic and functional diversity among mixotrophic consumers of Prochlorococcus. *ISME J* 2022;16:1557–69. 10.1038/s41396-022-01204-z PMC9122939 35145244

[16] Edwards KF, Li Q, Mcbeain KA, et al. Trophic strategies explain the ocean niches of small eukaryotic phytoplankton. *Proc R Soc B Biol Sci* 2023;290:20222021. 10.1098/rspb.2022.2021 PMC9874276 36695036

[17] Mitra A, Caron DA, Faure E, et al. The Mixoplankton database – diversity of photo-phago-trophic plankton in form, function and distribution across the global ocean. *J Eukaryot Microbiol* 2023;70:e12972–25. 10.1111/jeu.12972 36847544

[18] Larsson ME, Leles SG, Mitra A, et al. Environmental structuring of mixoplankton functional types within marine protist communities: a global analysis. *Ocean Microbiol* 2026;2:1. 10.1186/s44375-026-00007-3

[19] Jones EL, Menden-Deuer S, Rynearson TA. Dinoflagellate and ciliate trophic modes database. *Microbiol Resour Announc* 2025;14:e0015425. 10.1128/mra.00154-25 PMC12243557 40492775

[20] Calbet A . Cultured *versus* wild zooplankton and mixoplankton in experimental ecology: what we gain, what we lose and how to bridge the gap. *J Plankton Res* 2026;48:48. 10.1093/plankt/fbag008

[21] Costa MRA, Sarmento H, Becker V, et al. Phytoplankton phagotrophy across nutrients and light gradients using different measurement techniques. *J Plankton Res* 2022;44:507–20. 10.1093/plankt/fbac035

[22] Connell PE, Ribalet F, Armbrust EV, et al. Diel oscillations in the feeding activity of heterotrophic and mixotrophic nanoplankton in the North Pacific subtropical gyre. *Aquat Microb Ecol* 2020;85:167–81. 10.3354/AME01950

[23] Lambert BS, Groussman RD, Schatz MJ, et al. The dynamic trophic architecture of open-ocean protist communities revealed through machine-guided metatranscriptomics. *Proc Natl Acad Sci USA* 2022;119:e2100916119. 10.1073/pnas.2100916119 PMC8851463 35145022

[24] Thomas E, Groussman MJ, Coesel SN, et al. Latitude- and depth-driven divergence in protist trophic strategies revealed by a machine learning model. *Front Microbiol* 2025;16:1602162. 10.3389/fmicb.2025.1602162 PMC12434009 40959226

[25] Burns JA, Pittis AA, Kim E. Gene-based predictive models of trophic modes suggest Asgard archaea are not phagocytotic. *Nat Ecol Evol* 2018;2:697–704. 10.1038/s41559-018-0477-7 29459706

[26] Flynn KJ, Mitra A, Anestis K, et al. Mixotrophic protists and a new paradigm for marine ecology: where does plankton research go now? *J Plankton Res* 2019;41:375–91. 10.1093/plankt/fbz026

[27] Li H, Chen J, Yu L, et al. In situ community transcriptomics illuminates CO_2_ -fixation potentials and supporting roles of phagotrophy and proton pump in plankton in a subtropical marginal sea. *Microbiol Spectrum* 2024;12:02177–23. 10.1128/spectrum.02177-23 PMC10913738 38319114

[28] Kanehisa M, Goto S, Sato Y, et al. Data, information, knowledge and principle: back to metabolism in KEGG. *Nucleic Acids Res* 2014;42:D199–205. 10.1093/nar/gkt1076 PMC3965122 24214961

[29] Roden GI . Temperature and salinity fronts at the boundaries of the subarctic-subtropical transition zone in the western Pacific. *J Geophys Res* 1972;77:7175–87. 10.1029/JC077i036p07175

[30] Ribalet F, Berthiaume C, Hynes A, et al. SeaFlow data v1, high-resolution abundance, size and biomass of small phytoplankton in the North Pacific. *Sci Data* 2019;6:277. 10.1038/s41597-019-0292-2 PMC6874581 31757971

[31] Juranek LW, White AE, Dugenne M, et al. The importance of the phytoplankton “middle class” to ocean net community production. *Glob Biogeochem Cycles* 2020;34:e2020GB006702. 10.1029/2020GB006702

[32] Key RS, Coesel SN, Gradoville MR, et al. Picophytoplankton implicated in productivity and biogeochemistry in the North Pacific transition zone. *mSystems* 2026;11:e0080125. 10.1128/msystems.00801-25 PMC12911361 41543250

[33] Groussman RD, Coesel SN, Durham BP, et al. The North Pacific eukaryotic gene catalog of metatranscriptome assemblies and annotations. *Sci Data* 2024;11:1161. 10.1038/s41597-024-04005-5 PMC11496615 39438508

[34] Ashkezari MD, Hagen NR, Denholtz M, et al. Simons collaborative marine atlas project (Simons CMAP): an open-source portal to share, visualize, and analyze ocean data. *Limnol Oceanogr Methods* 2021;19:488–96. 10.1002/lom3.10439

[35] Foreman RK, Barone B, Grabowski E, et al. Biogeochemical anatomy and ecosystem dynamics of a large phytoplankton bloom north of the Hawaiian islands. *Prog Oceanogr* 2026;241:103620. 10.1016/j.pocean.2025.103620

[36] Letelier RM, Karl DM, Abbott MR, et al. Light driven seasonal patterns of chlorophyll and nitrate in the lower euphotic zone of the North Pacific subtropical gyre. *Limnol Oceanogr* 2004;49:508–19. 10.4319/lo.2004.49.2.0508

[37] Groussman RD, Blaskowski S, Coesel SN, et al. MarFERReT, an open-source, version-controlled reference library of marine microbial eukaryote functional genes. *Sci Data* 2023;10:926. 10.1038/s41597-023-02842-4 PMC10739892 38129449

[38] Grabherr MG, Haas BJ, Yassour M, et al. Full-length transcriptome assembly from RNA-seq data without a reference genome. *Nat Biotechnol* 2011;29:644–52. 10.1038/nbt.1883 PMC3571712 21572440

[39] Buchfink B, Xie C, Huson DH. Fast and sensitive protein alignment using DIAMOND. *Nat Methods* 2015;12:59–60. 10.1038/nmeth.3176 25402007

[40] Aramaki T, Blanc-Mathieu R, Endo H, et al. KofamKOALA: KEGG ortholog assignment based on profile HMM and adaptive score threshold. *Bioinformatics* 2020;36:2251–2. 10.1093/bioinformatics/btz859 PMC7141845 31742321

[41] Coesel SN, Graff van Creveld S, Dugenne M, et al. Proportional relationship between transcript concentrations and carbon biomass for open ocean plankton groups. *ISME J* 2025;19:19. 10.1093/ismejo/wraf079 PMC12085914 40302033

[42] Satinsky BM , Gifford SM, Crumb BC, et al. Use of internal standards for quantitative Metatranscriptome and metagenome analysis. In: DeLong EF, ed. Methods in Enzymology, Vol. 531. New York: Elsevier, 2013, 237–50. 10.1016/B978-0-12-407863-5.00012-5. 24060124

[43] Glynn EF, Chen J, Mushegian AR. Detecting periodic patterns in unevenly spaced gene expression time series using Lomb–Scargle periodograms. *Bioinformatics* 2006;22:310–6. 10.1093/bioinformatics/bti789 16303799

[44] Wilson ST, Aylward FO, Ribalet F, et al. Coordinated regulation of growth, activity and transcription in natural populations of the unicellular nitrogen-fixing cyanobacterium Crocosphaera. *Nat Microbiol* 2017;2:17118. 10.1038/nmicrobiol.2017.118 28758990

[45] Groussman RD, Coesel SN, Durham BP, et al. Diel-regulated transcriptional cascades of microbial eukaryotes in the North Pacific subtropical gyre. *Front Microbiol* 2021;12:1–15. 10.3389/fmicb.2021.682651 PMC8511712 34659137

[46] Graff van Creveld S, Coesel SN, Schatz MJ, et al. New eukaryotic phytoplankton isolates from the oligotrophic tropical Pacific Ocean. *J Phycol* 2024;60:598–603. 10.1111/jpy.13447 38625734

[47] Dodd AN, Kusakina A, Hall A, et al. The circadian regulation of photosynthesis. *Photosynth Res* 2014;119:181–90. 10.1007/s11120-013-9811-8 23529849

[48] Zaheri B, Morse D. An overview of transcription in dinoflagellates. *Gene* 2022;829:146505. 10.1016/j.gene.2022.146505 35447242

[49] Coesel SN, Durham BP, Groussman RD, et al. Diel transcriptional oscillations of light-sensitive regulatory elements in open-ocean eukaryotic plankton communities. *Proc Natl Acad Sci USA* 2021;118:e2011038118. 10.1073/pnas.2011038118 PMC8017926 33547239

[50] Rees TAV, Raven JA. The maximum growth rate hypothesis is correct for eukaryotic photosynthetic organisms, but not cyanobacteria. *New Phytol* 2021;230:601–11. 10.1111/nph.17190 PMC8048539 33449358

[51] Guillou L, Bachar D, Audic S, et al. The protist ribosomal reference database (PR2): a catalog of unicellular eukaryote small sub-unit rRNA sequences with curated taxonomy. *Nucleic Acids Res* 2012;41:D597–604. 10.1093/nar/gks1160 PMC3531120 23193267

[52] Izett RW, Fennel K, Stoer AC, et al. Reviews and syntheses: expanding the global coverage of gross primary production and net community production measurements using biogeochemical-Argo floats. *Biogeosciences* 2024;21:13–47. 10.5194/bg-21-13-2024

[53] Nair A, Sathyendranath S, Platt T, et al. Remote sensing of phytoplankton functional types. *Remote Sens Environ* 2008;112:3366–75. 10.1016/j.rse.2008.01.021

[54] Serret P, Robinson C, Aranguren-Gassis M, et al. Both respiration and photosynthesis determine the scaling of plankton metabolism in the oligotrophic ocean. *Nat Commun* 2015;6:6961. 10.1038/ncomms7961 PMC4462842 25908109

[55] Becker KW, Harke MJ, Mende DR, et al. Combined pigment and metatranscriptomic analysis reveals highly synchronized diel patterns of phenotypic light response across domains in the open oligotrophic ocean. *ISME J* 2021;15:520–33. 10.1038/s41396-020-00793-x PMC8027880 33033374

[56] Calbet A, Landry MR. Phytoplankton growth, microzooplankton grazing, and carbon cycling in marine systems. *Limnol Oceanogr* 2004;49:51–7. 10.4319/lo.2004.49.1.0051

[57] Behrenfeld MJ, Boss ES. Resurrecting the ecological underpinnings of ocean plankton blooms. *Annu Rev Mar Sci* 2014;6:167–94. 10.1146/annurev-marine-052913-021325 24079309

[58] Benoiston A-S, Ibarbalz FM, Bittner L, et al. The evolution of diatoms and their biogeochemical functions. *Philos Trans R Soc B Biol Sci* 2017;372:20160397. 10.1098/rstb.2016.0397 PMC5516106 28717023

[59] Graff van Creveld S, Rosenwasser S, Levin Y, et al. Chronic iron limitation confers transient resistance to oxidative stress in marine diatoms. *Plant Physiol* 2016;172:00840.2016–979. 10.1104/pp.16.00840 PMC5047098 27503604

[60] Lomas MW, Glibert PM. Temperature regulation of nitrate uptake: a novel hypothesis about nitrate uptake and reduction in cool-water diatoms. *Limnol Oceanogr* 1999;44:556–72. 10.4319/lo.1999.44.3.0556

[61] Joli N, Concia L, Mocaer K, et al. Hypometabolism to survive the long polar night and subsequent successful return to light in the diatom *Fragilariopsis cylindrus*. *New Phytol* 2024;241:2193–208. 10.1111/nph.19387 38095198

[62] Li Q, Edwards KF, Schvarcz CR, et al. Plasticity in the grazing ecophysiology of *Florenciella* (Dichtyochophyceae), a mixotrophic nanoflagellate that consumes *Prochlorococcus* and other bacteria. *Limnol Oceanogr* 2021;66:47–60. 10.1002/lno.11585

[63] Hagino K, Onuma R, Kawachi M, et al. Discovery of an endosymbiotic nitrogen-fixing cyanobacterium UCYN-A in *Braarudosphaera bigelowii* (Prymnesiophyceae). *PLoS One* 2013;8:8. 10.1371/journal.pone.0081749 PMC3852252 24324722

[64] Coale TH, Loconte V, Turk-Kubo KA, et al. Nitrogen-fixing organelle in a marine alga. *Science* 2024;384:217–22. 10.1126/science.adk1075 38603509

[65] Pierella Karlusich JJ, Ibarbalz FM, Bowler C. Exploration of marine phytoplankton: from their historical appreciation to the omics era. *J Plankton Res* 2020;42:595–612. 10.1093/plankt/fbaa049

[66] Ward BA, Dutkiewics S, Jahn O, et al. A size-structured food-web model for the global ocean. *Limnol Oceanogr* 2012;57:1877–91. 10.4319/lo.2012.57.6.1877

[67] Dutkiewicz S , Follet CL, Follows MJ, et al. Multiple biotic interactions establish phytoplankton community structure across environmental gradients. *Limnol Oceanogr* 2024;69:1086–100. 10.1002/lno.12555

