# Supplementary material for: Balancing photosynthesis and respiration in North Pacific protist communities
Source: ISME J. 2026 Sep 11;20(1):wrag234. doi: 10.1093/ismejo/wrag234 (PMC13637818; doi:10.1093/ismejo/wrag234)
Supplement: Supplementary file 1 [file supplementary_material_wrag234.zip › Suppl_Figures_tables_TiP_Coesel_R1.pdf]

Supplemental Information for:

Balancing photosynthesis and respiration in North Pacific plankton communities

'Protist energy allocation'

Sacha N. Coesel[1*], Shiri Graff van Creveld[1], Mathilde Dugenne[2], Bryndan P. Durham[3], Michael J. Follows[4], Rebecca S. Key[3], Francois Ribalet[1], Angelicque E. White[2], E. Virginia Armbrust[1].

[1] School of Oceanography, University of Washington, Seattle, WA 98195, USA

[2] Department of Oceanography, University of Hawai'i at Manoa, Honolulu, HI 96822, USA

[3] Department of Biology, Genetics Institute, University of Florida, Gainesville, Florida, USA

[4] Center for Global Change Sciences, Massachusetts Institute of Technology, Cambridge MA, USA

[*]Corresponding author: Sacha N. Coesel. School of Oceanography, University of Washington, Seattle, WA 98195, USA. Email: coesel@uw.edu

This document contains:

Page 1: Front page

Page 2-18: Supplemental Figures S1-S16

Page 19-21: Supplemental Tables S1-S3

Page 22-26: Information for Supplemental Datasheet S1-S5

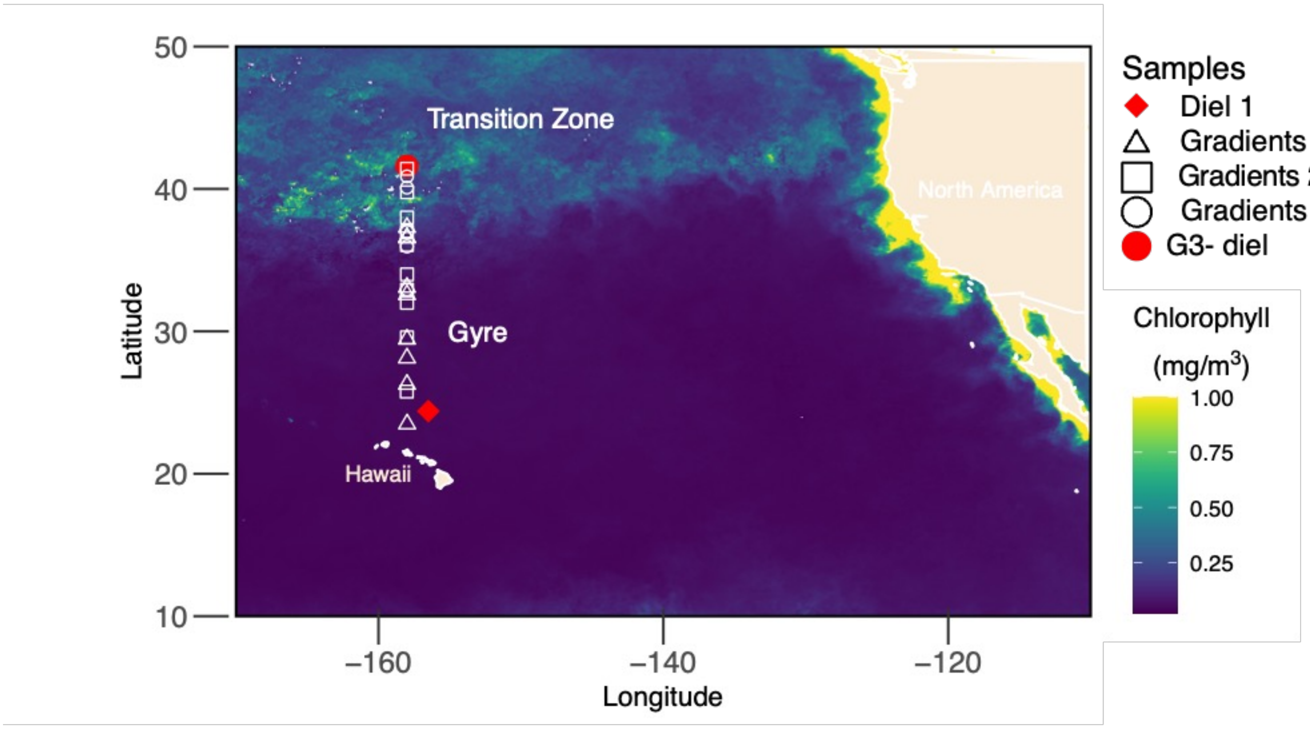

Figure S1. Sampling sites for metatranscriptomes in the North Pacific Gyre and TZ. Latitudinal locations along 158° N of the Gradients 1, 2, and 3 transects, as well as the multi-day D1-diel and G3-diel datasets, shown against a climatology of satellite-derived chlorophyll concentrations for May-June 2017 (G2). NASA MODIS Aqua 8-day averaged chlorophyll data were retrieved from Simons CMAP (https://simonscmap.com) [34].

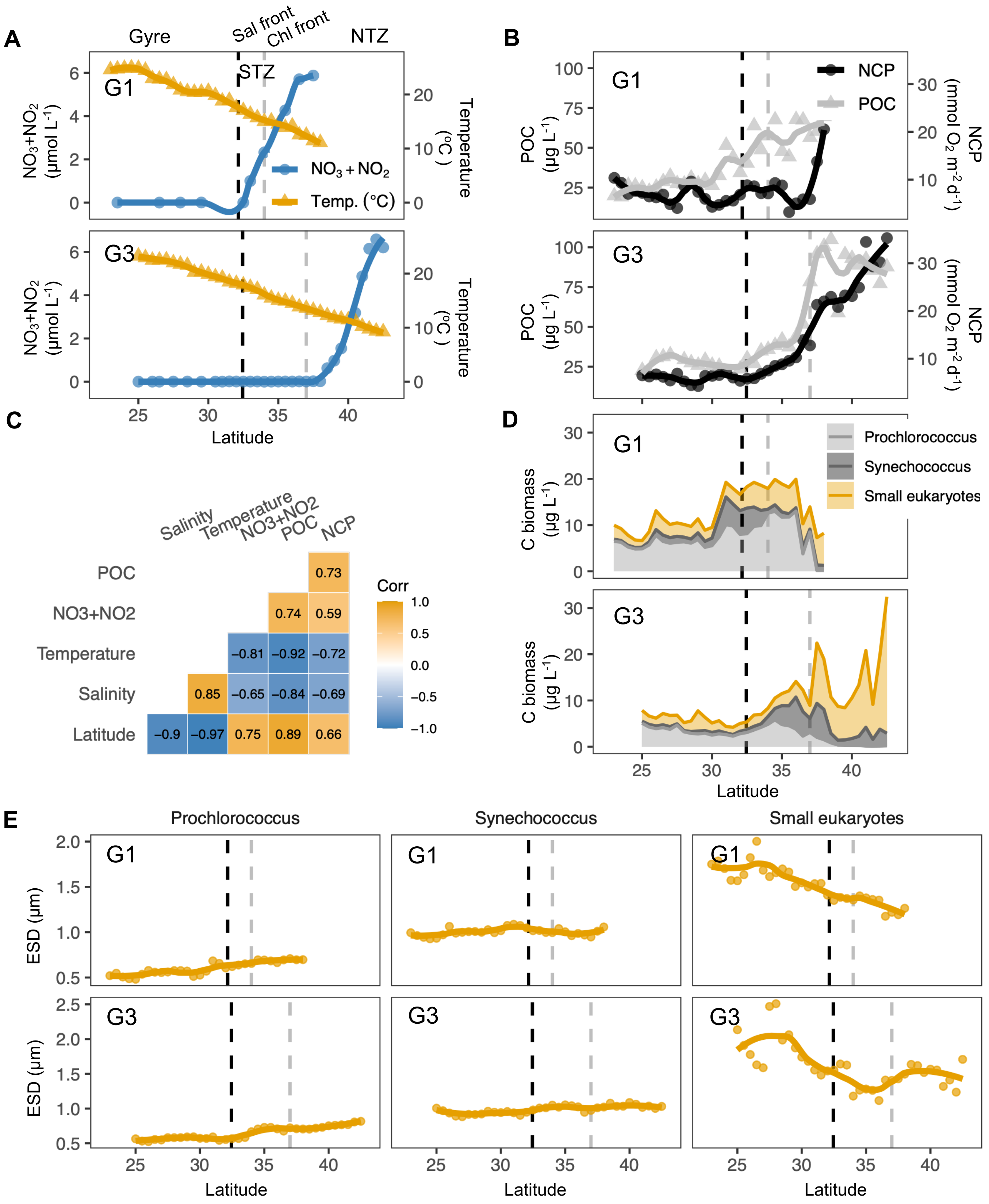

Figure S2. Biogeochemical and plankton structure along the Gradients 1 and 3 transect. (A) Surface water temperature (yellow) and inorganic nitrate concentration (blue) plotted against latitude (°N). The vertical dashed black lines represent the 34.82 isohaline, marking the boundary between the North Pacific Subtropical Gyre and the Southern Transition Zone (STZ). *Cont. next page*

Legend S2 cont. The dashed grey lines indicate the 0.2 mg m^{-3} Chlorophyll a threshold, delineating the boundary with the Northern Transition Zone (NTZ). (B) Net Community Productivity (NCP; black), derived from O_2/Argon ratios, and particulate organic carbon (POC; grey) concentrations from discrete samples. (C) Spearman correlations of environmental variables, POC and NCP. Only correlations with $P < 0.01$ are shown. (D) Estimated carbon biomass concentrations (mean per 0.5° latitude) of picoplankton, including *Prochlorococcus*, *Synechococcus* (Cyanobacteria), and small eukaryotes (0.2-5 µm) from continuous flow cytometry (SeaFlow) [30]. (E) Median equivalent spherical diameter (ESD) per 0.5° latitude for *Prochlorococcus*, *Synechococcus*, and small eukaryotes. The orange line represents a loess smoothing fit of the ESD (span = 0.5).

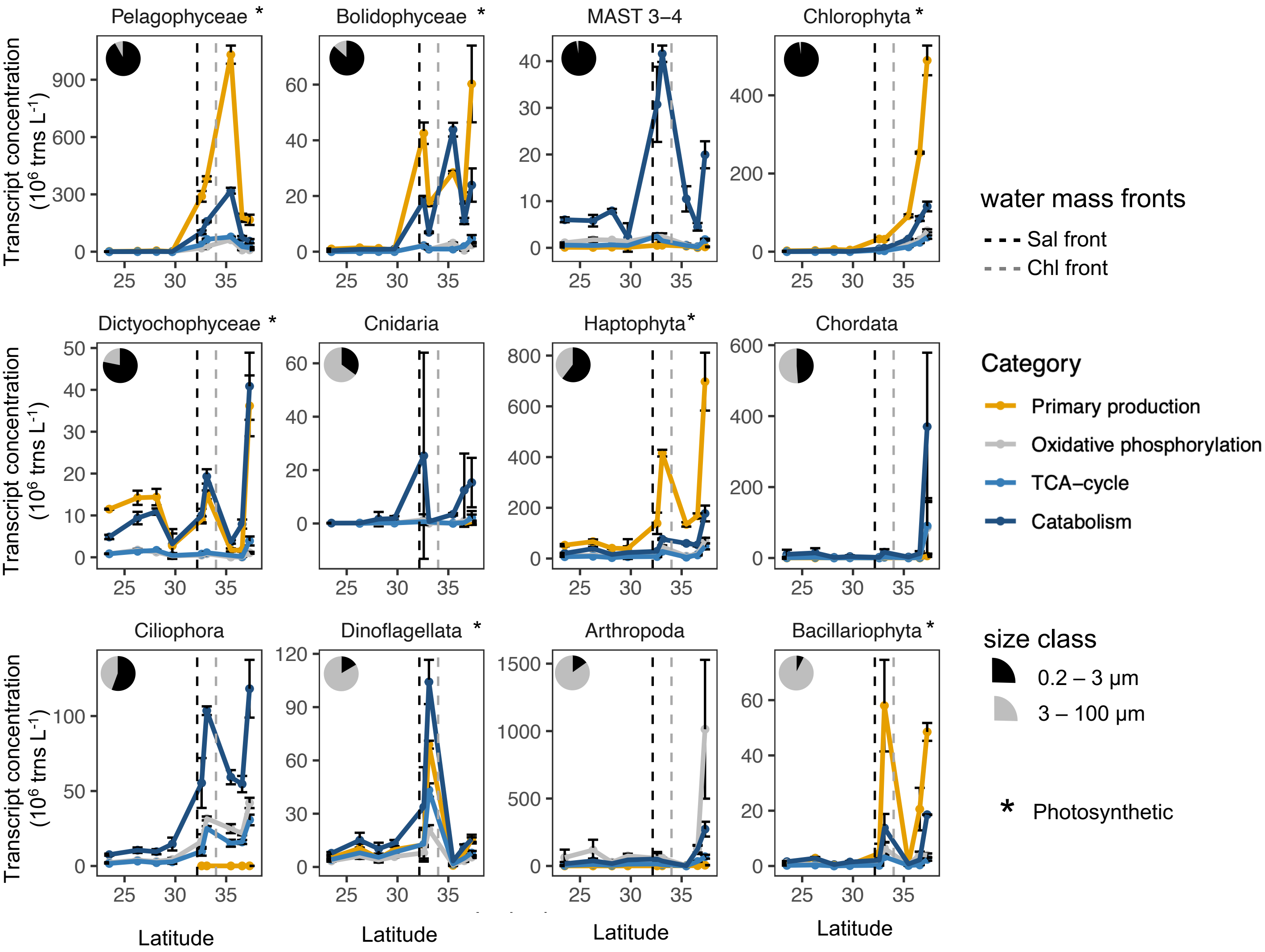

Figure S3. Gradients 1 (2016) photosynthesis and respiration transcript abundance for the 12 most abundant plankton taxa. Line plots show transcript concentrations (10^9 transcripts L^{-1}) related to photosynthesis, nitrate assimilation, oxidative phosphorylation, TCA cycle, and catabolism (see also Table 1, Datasheet S1). Data represent the 12 most abundant plankton taxa, with mean ± SD from biological triplicates. Transcripts from small (0.2-3 µm) and large (3-100 µm) filter size fractions are combined. Pie charts show the proportion of transcripts from the small (black) vs. large (grey) fraction. Dashed lines indicate environmental fronts: black = salinity, grey = chlorophyll *a*. Asterisks (*) indicate detection of photosynthesis-related genes in the respective group. Dinoflagellate transcript levels are scaled by 1/6.4 to account for taxon-specific transcript-to-biomass conversion [41].

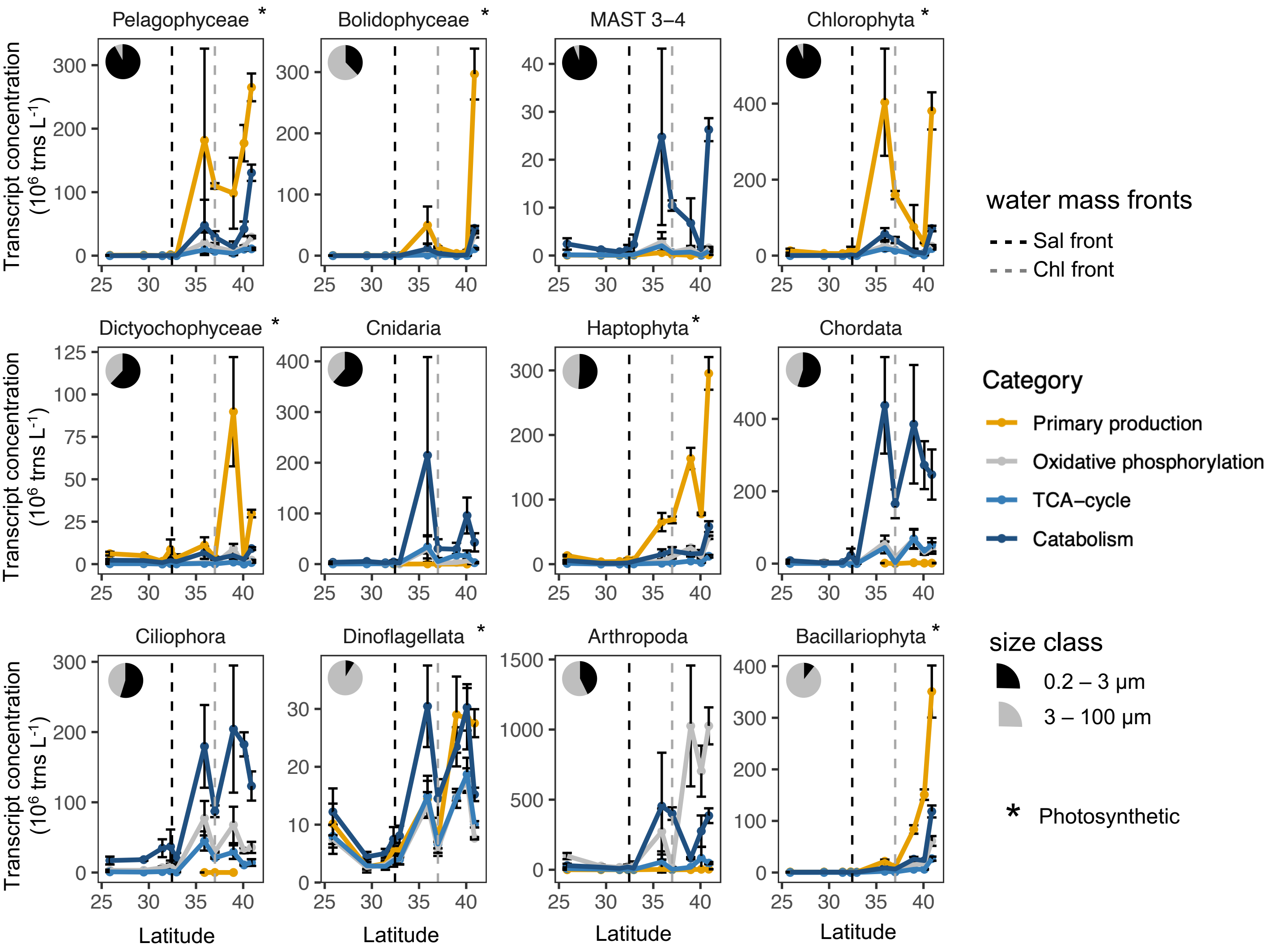

Figure S4. Gradients 3 (2019) photosynthesis and respiration transcript abundance for the 12 most abundant plankton taxa Line plots show transcript concentrations (10^9 transcripts L^{-1}) related to photosynthesis, nitrate assimilation, oxidative phosphorylation, TCA cycle, and catabolism (see also Table 1). Data represent the 12 most abundant plankton taxa, with mean ± SD from biological triplicates. Transcripts from small (0.2-3 µm) and large (3-100 µm) filter size fractions are combined. Pie charts show the proportion of transcripts from the small (black) vs. large (grey) fraction. Dashed lines indicate environmental fronts: black = salinity, grey = chlorophyll *a*. Asterisks (*) indicate detection of photosynthesis-related genes in the respective group. Dinoflagellate transcript levels are scaled by 1/6.4 to account for taxon-specific transcript-to-biomass conversion [41].

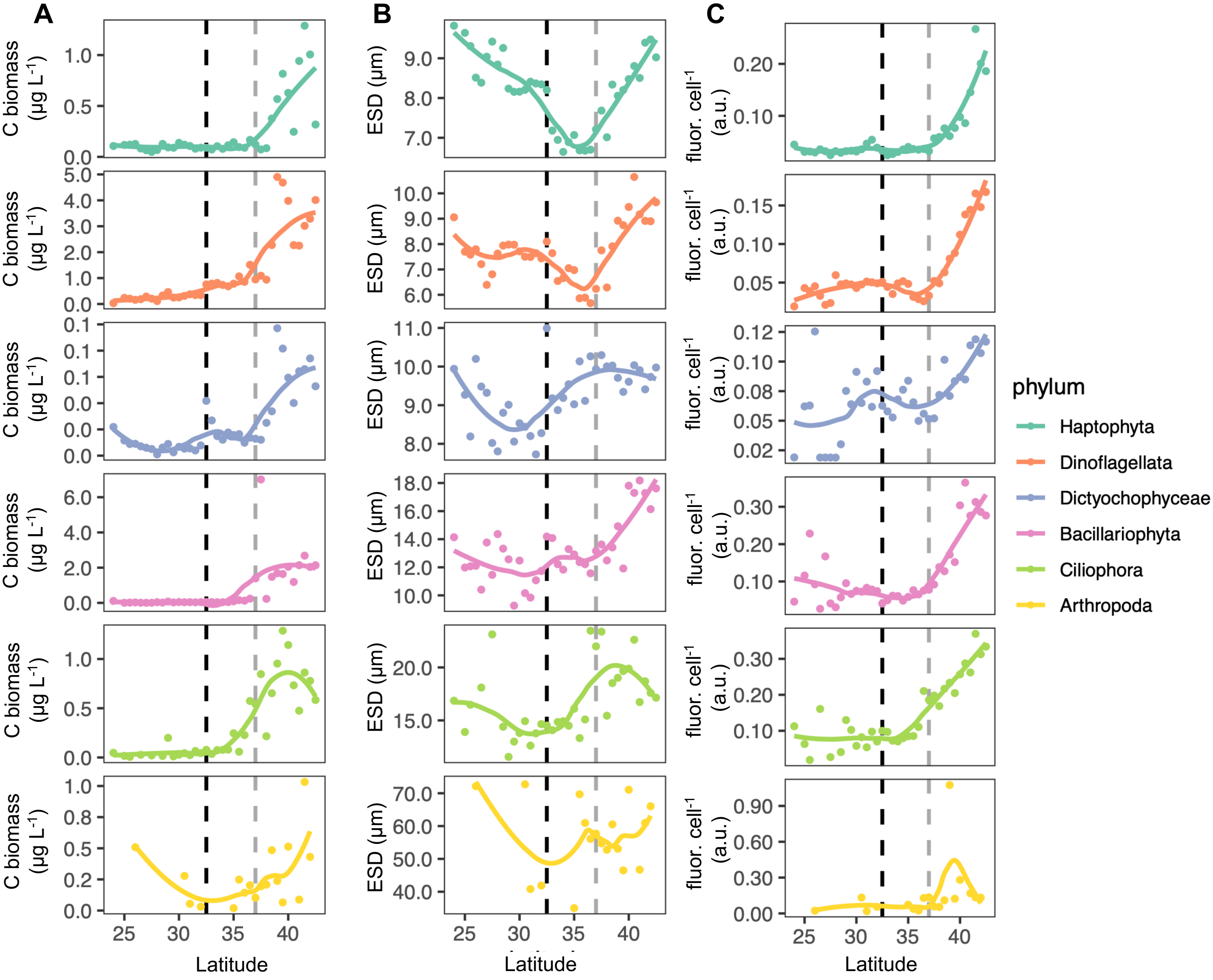

Figure S5. Phylum/class-level latitudinal trends in IFCb-derived metrics of dominant large-size eukaryotic plankton along Gradients 2. A) Carbon biomass concentrations at the phylum/class level, averaged within 0.5° latitude bins. (B) Median equivalent spherical diameter (ESD) and metatranscriptome-derived TiP ratios (mean ± SD; black) for the large size fraction, shown at the phylum/class level. (C) Peak fluorescence per cell (arbitrary units), averaged within 0.5° latitude bins. The lines represents loess smoothing fits (span = 0.6). Dashed black and grey lines indicate the salinity and chlorophyll fronts, respectively.

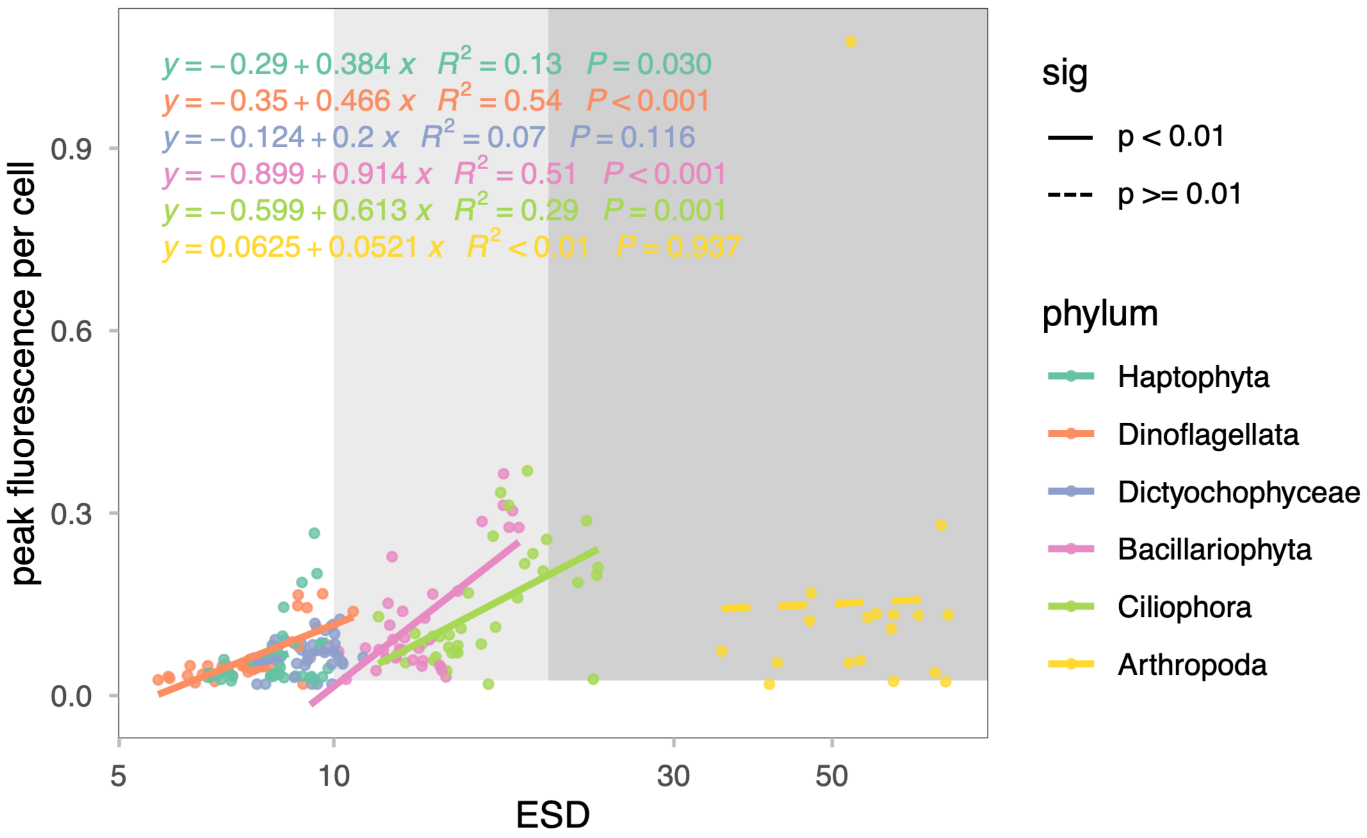

Figure S6. Relationship between cellular peak fluorescence and cell size. Scatterplot of IFCb-derived peak fluorescence per cell (arbitrary units) versus IFCb-derived equivalent spherical diameter (μm) for each phylum/class. Data were averaged within 0.5° latitude bins along the Gradient 2 transect. The light grey shaded area denotes the nanoplankton size range (10–20 μm ESD), and the dark grey shaded area denotes the microplankton size range (>20 μm ESD). Linear regression lines are shown together with the corresponding regression equations, R^2 values, and *p*-values. Solid lines indicate significant relationships *(P* < 0.01), whereas dashed lines indicate non-significant relationships ($P \geq 0.01$).

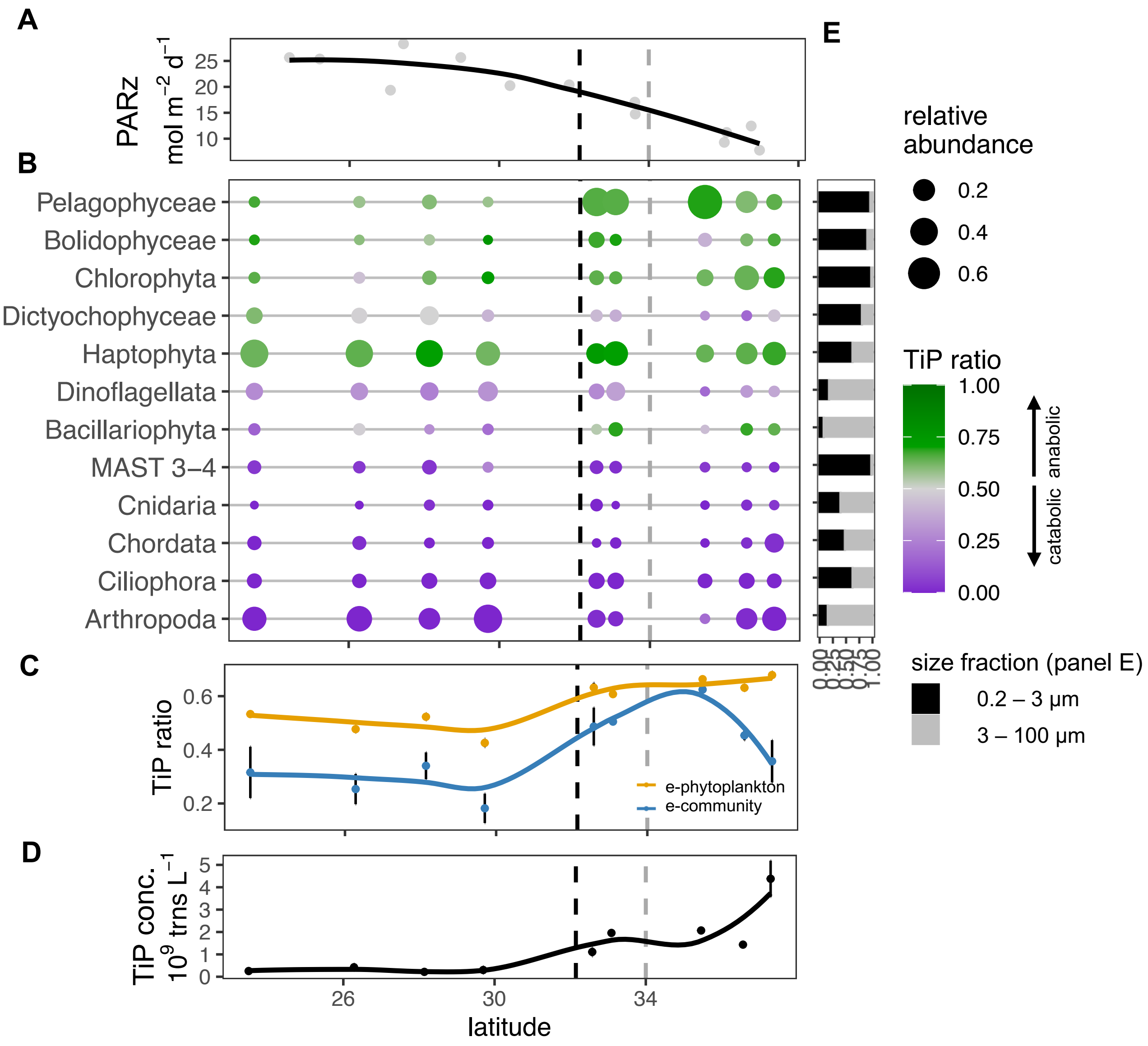

Figure S7. Gradients 1 latitudinal trends in relative transcript abundance and TiP of eukaryotic plankton. (A) Daily-integrated Surface Photosynthetically Active Radiation (PAR) estimated per 1 degree latitude. (B) Relative abundance (bubble size) and TiP ratio (color gradient) of eukaryotic phytoplankton and zooplankton. Relative abundance is calculated as ratio to total TiP-related transcripts. The TiP ratio reflects the ratio of transcripts associated with photosynthesis relative to the sum of photosynthesis and respiration transcripts combined (see Table 1). Samples collected at 04:00h. Dinoflagellate values were scaled by a factor of 1/6.4 based on transcript-to-biomass estimates [41]. (C) Community-level TiP ratios (combined top 12 taxa) and Phototrophs (combined photosynthesizing phyla) , with error bars representing the standard error of the mean. D) Estimated transcript concentrations (TiP transcripts only) of the combined top12 taxa. Dashed black and grey lines denote the salinity and chlorophyll fronts, respectively. (E) Transcript distributions across small and large size fractions.

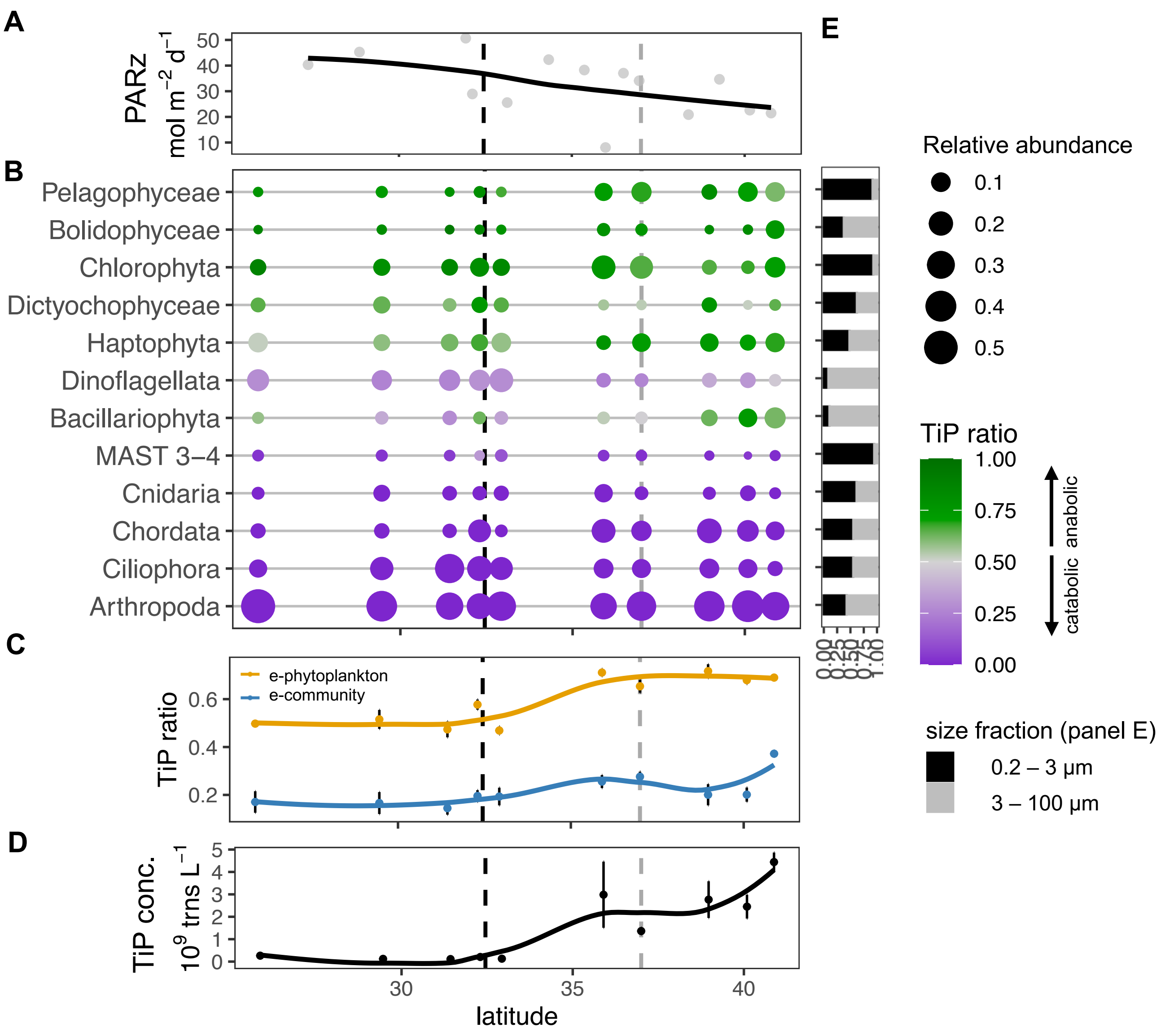

Figure S8. Gradients 3 latitudinal trends in relative transcript abundance and TiP of eukaryotic plankton. (A) Daily-integrated Surface Photosynthetically Active Radiation (PAR) estimated per 1 degree latitude. (B) Relative abundance (bubble size) and TiP ratio (color gradient) of eukaryotic phytoplankton and zooplankton. Relative abundance is calculated as ratio to total TiP-related transcripts. The TiP ratio reflects the ratio of transcripts associated with photosynthesis relative to the sum of photosynthesis and respiration transcripts combined (see Table 1). Samples collected at 04:00h. Dinoflagellate values were scaled by a factor of 1/6.4 based on transcript-to-biomass estimates [41]. (C) Community-level TiP ratios (combined top 12 taxa) and Phototrophs (combined photosynthesizing phyla) , with error bars representing the standard error of the mean. D) Estimated transcript concentrations (TiP transcripts only) of the combined top12 taxa. Dashed black and grey lines denote the salinity and chlorophyll fronts, respectively. (E) Transcript distributions across small and large size fractions.

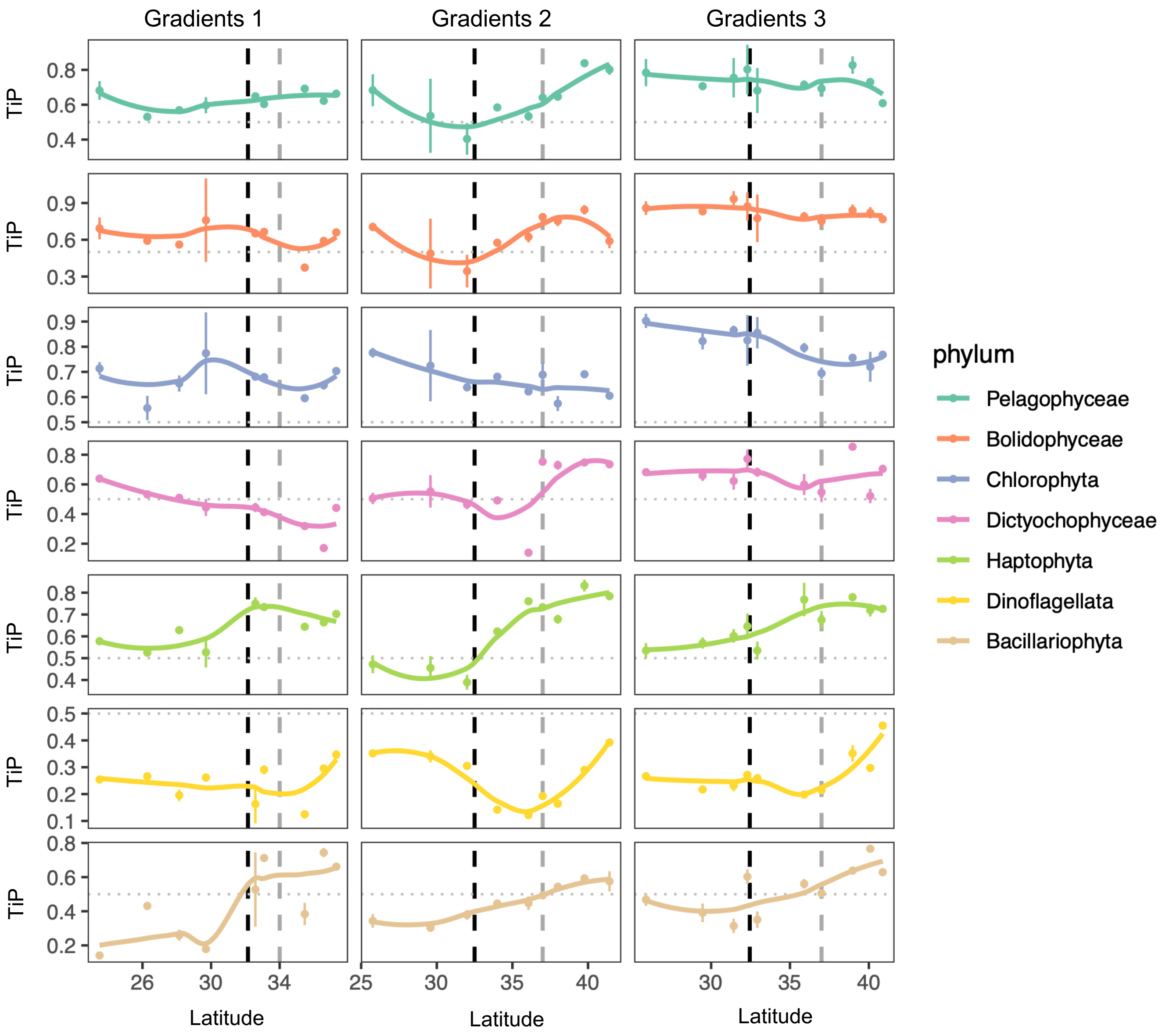

Figure S9. Latitudinal trends in TiP of dominant eukaryotic phytoplankton taxa along Gradients 1~3. Line plots show TiP ratios for the 12 most abundant plankton taxa plotted against latitude (°N), with means ± SD from biological triplicates. The TiP ratio represents the proportion of transcripts associated with photosynthesis relative to the combined total of photosynthesis and respiration transcripts (see Table 1). Samples were collected at 06:00h for Gradients 1 and 3 and at 04:00 h for Gradient 2. The lines represents loess smoothing fits (span = 0.5). Dashed vertical lines indicate environmental fronts (black = salinity; grey = chlorophyll *a*).

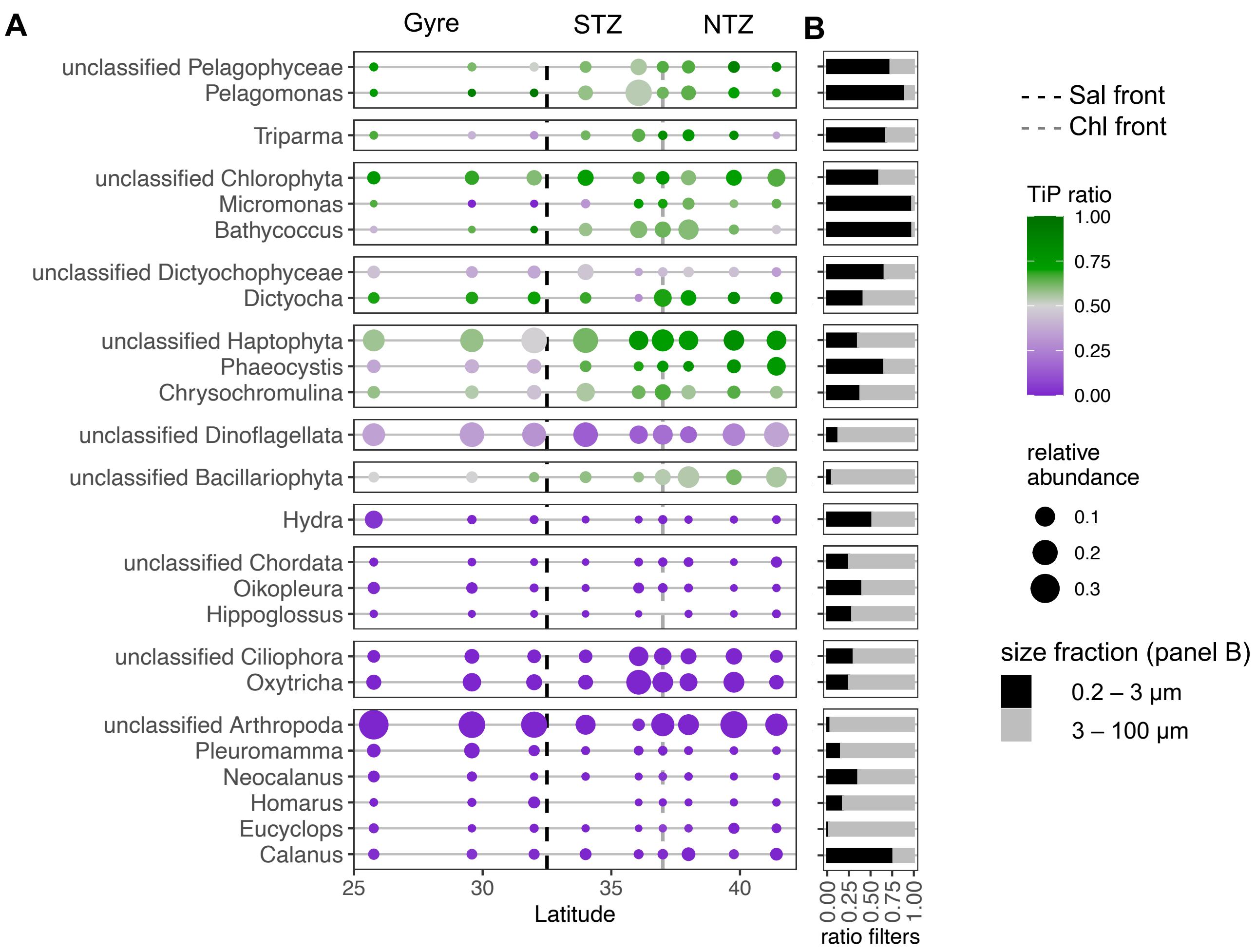

Figure S10. Gradients 2 relative transcript abundance and TiP of eukaryotic plankton at genus level. (A) Relative abundance (bubble size) and TiP ratio (color gradient) of eukaryotic plankton annotated at the genus level across the Gradients 2 transect (samples collected at 04:00h). Relative abundance is calculated as ratio to total top12 TiP transcripts. The TiP ratio reflects the ratio of transcripts associated with photosynthesis relative to the sum of photosynthesis and respiration transcripts combined (see Table 1). Only taxa contributing ≥5% of transcripts are included; "Unclassified" refers to transcripts without genus-level identification. Dinoflagellate values were scaled by a factor of 1/6.4 based on transcript-to-biomass estimates [41]. (B) Transcript distributions across small and large size fractions are displayed. Dashed black and grey lines denote the salinity and chlorophyll fronts, respectively.

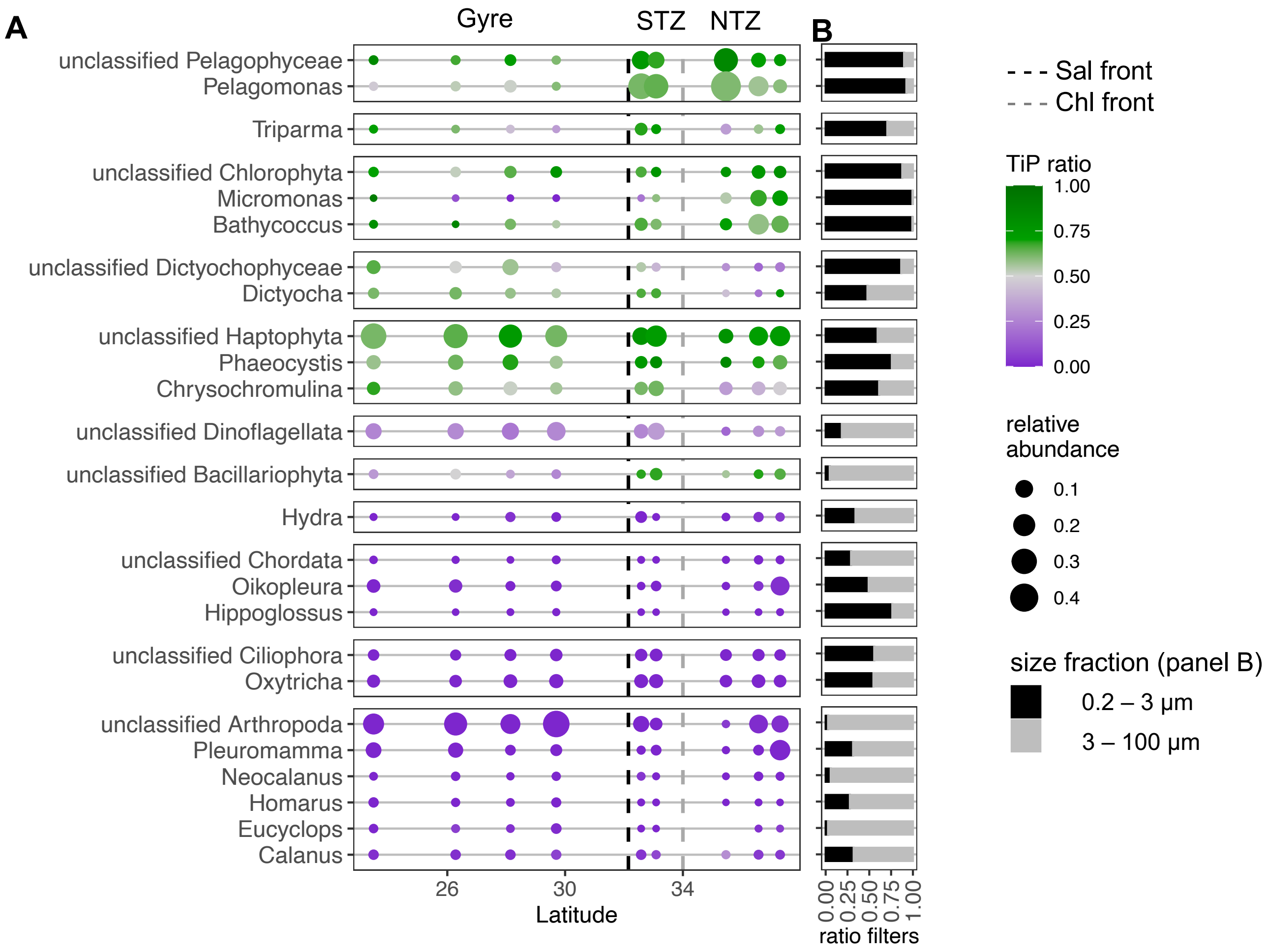

Figure S11. Gradients 1 relative transcript abundance and TiP of eukaryotic plankton at genus level. (A) Relative transcript abundance (bubble size) and TiP ratio (color gradient) of eukaryotic plankton annotated at the genus level across the Gradients 1 transect (samples collected at 06:00h). Relative abundance is calculated as ratio to total top12 TiP transcripts. The TiP ratio reflects the ratio of transcripts associated with photosynthesis relative to the sum of photosynthesis and respiration transcripts combined (see Table 1). Only taxa contributing ≥5% of transcripts are included; “Unclassified” refers to transcripts without genus-level identification. Dinoflagellate values were scaled by a factor of 1/6.4 based on transcript-to-biomass estimates [41]. (B) Transcript distributions across small and large size fractions are displayed. Dashed black and grey lines denote the salinity and chlorophyll fronts, respectively.

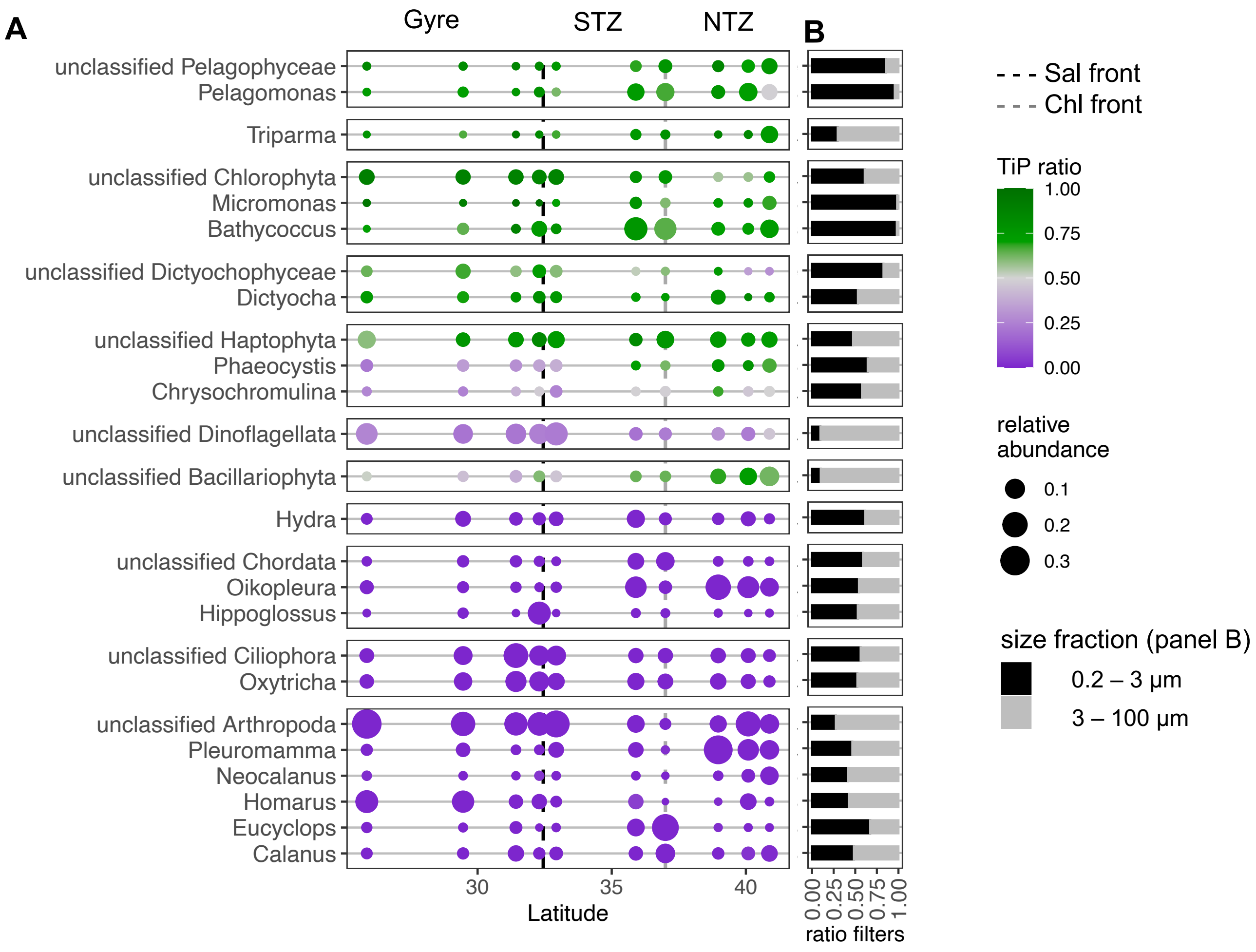

Figure S12. Gradients 3 relative transcript abundance and TiP of eukaryotic plankton at genus level. (A) Relative transcript abundance (bubble size) and TiP ratio (color gradient) of eukaryotic plankton annotated at the genus level across the Gradients 3 transect (samples collected at 06:00h). Relative abundance is calculated as ratio to total top12 TiP transcripts. The TiP ratio reflects the ratio of transcripts associated with photosynthesis relative to the sum of photosynthesis and respiration transcripts combined (see Table 1). Only taxa contributing ≥5% of transcripts are included; "Unclassified" refers to transcripts without genus-level identification. Dinoflagellate values were scaled by a factor of 1/6.4 based on transcript-to-biomass estimates [41]. (B) Transcript distributions across small and large size fractions are displayed. Dashed black and grey lines denote the salinity and chlorophyll fronts, respectively.

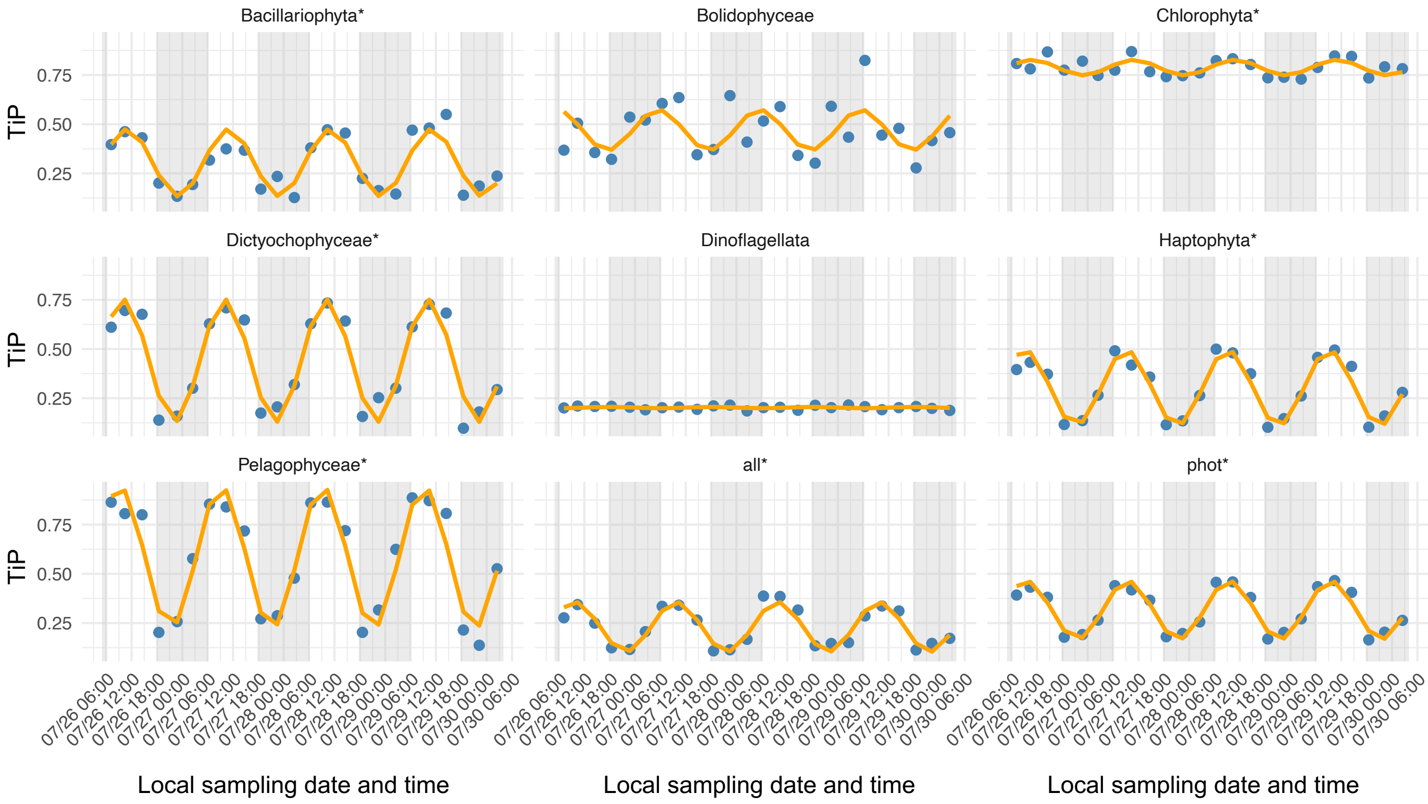

Figure S13. Assessment of diel periodicity of TiP for Diel1 (2015). Phytoplankton TiP ratios measured during the Diel1 cruise (July 2015; 24.4°N, 156°W). The TiP ratio represents the proportion of transcripts associated with photosynthesis relative to the combined total of photosynthesis and respiration transcripts. Samples were collected approximately every 4 hours during Lagrangian drifter deployments. Blue points show mean TiP values from biological triplicates. Solid lines indicate 24 h cosinor model fits used to visualize diel patterns. Shown are phytoplankton TiP at phylum/class level, as well as community-level TiP (combined phytoplankton and zooplankton; "all"), and phytoplankton-level TiP (combined phytoplankton; "phot"). Asterisks (*) identify taxa exhibiting significant diel periodicity ($p < 0.05$) based on Lomb-Scargle periodogram analysis [43]. Grey shaded regions denote nighttime.

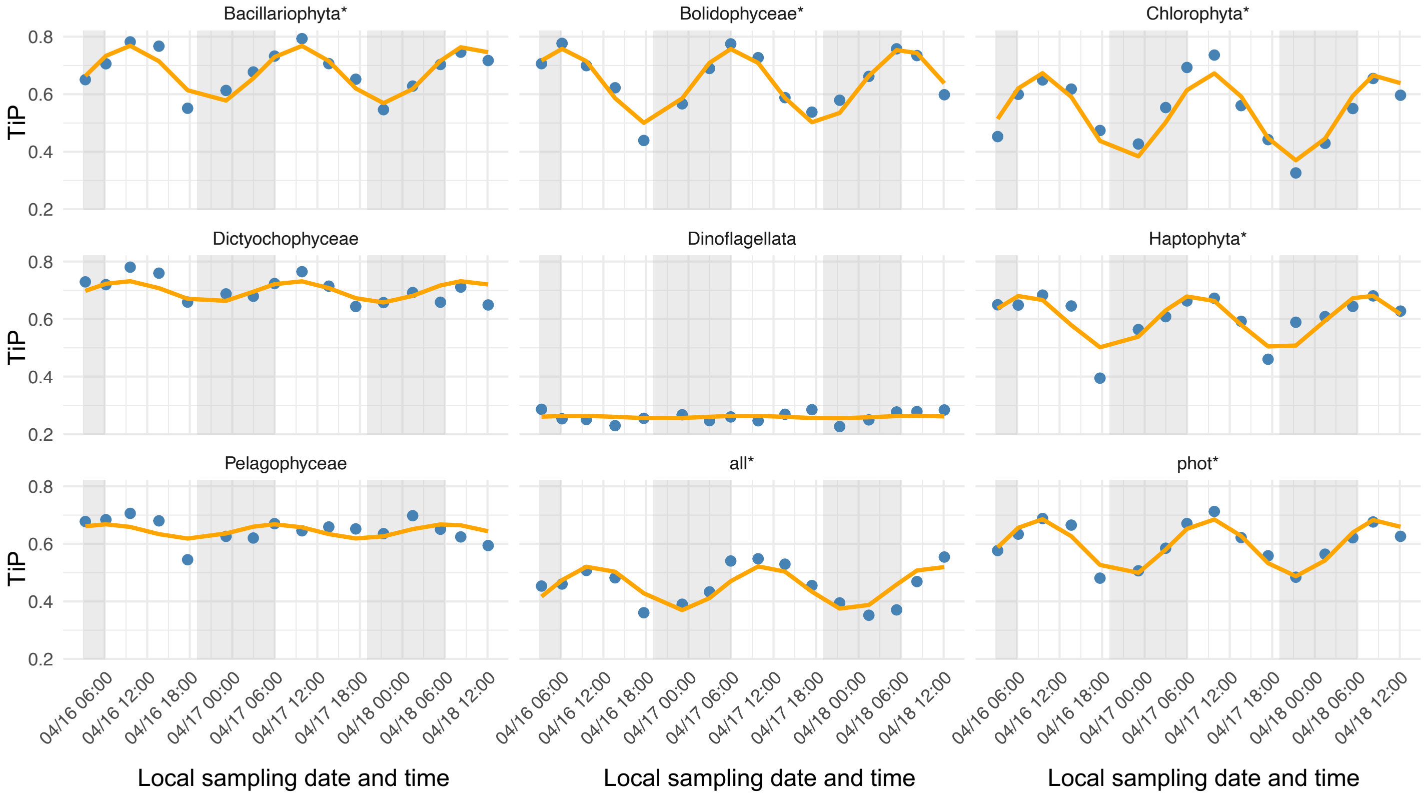

Figure S14. Assessment of diel periodicity of TiP for G3-Diel (2019) . Phytoplankton TiP ratios measured during the G3-diel cruise (April 2019, 41.6°N, 158°W). The TiP ratio represents the proportion of transcripts associated with photosynthesis relative to the combined total of photosynthesis and respiration transcripts. Samples were collected approximately every 4 hours during Lagrangian drifter deployments. Blue points show mean TiP values from biological triplicates. Solid lines indicate 24 h cosinor model fits used to visualize diel patterns. Shown are phytoplankton TiP at phylum/class level, as well as community-level TiP (combined phytoplankton and zooplankton; “all”), and phytoplankton-level TiP (combined phytoplankton; “phot”). Asterisks (*) identify taxa exhibiting significant diel periodicity ($p < 0.05$) based on Lomb-Scargle periodogram analysis [43]. Grey shaded regions denote nighttime.

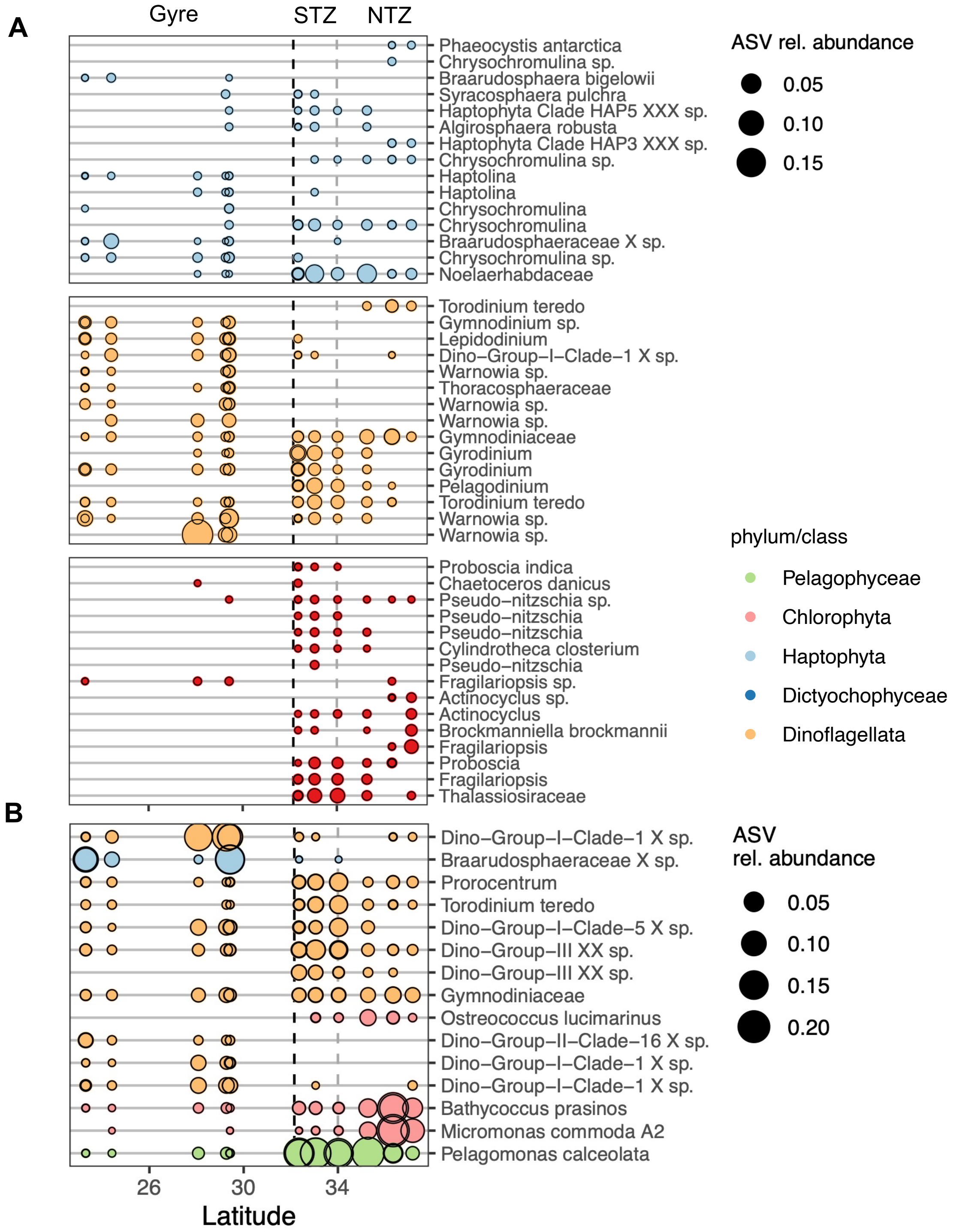

Figure S15. ASV-level taxonomic successions of eukaryotic phytoplankton along Gradients 1. (A) Large size fraction (3-100 µm): *Haptophyta* (top), *Dinoflagellata* (middle), and *Bacillariophyta* (bottom), showing the 15 most abundant and unique ASVs per taxonomic group. (B) Small size fraction (0.2-3 µm): top 15 ASVs of primary producers. Bubble plots represent ASVs (18S rRNA V4-V5 region), with bubble size proportional to relative abundance per site, calculated within each size fraction [32]. Taxonomies were assigned using PR2 v5.1 at the most precise rank supported by the sequence [32]. ASVs were ordered using hierarchical clustering (Ward's method; ward.D). ASV feature IDs and taxon strings are given in Datasheet S5.

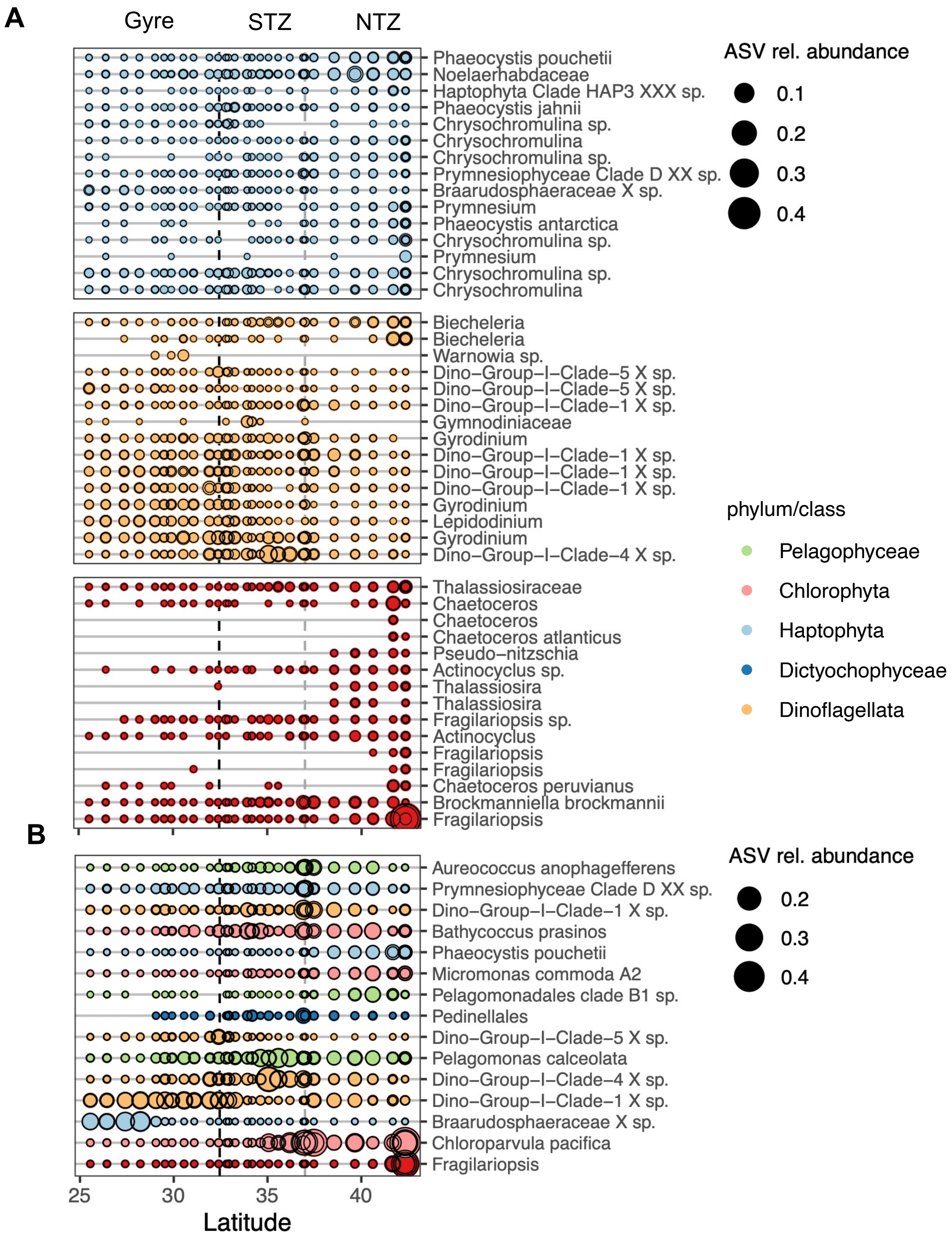

Figure S16. ASV-level taxonomic successions of eukaryotic phytoplankton along Gradients 3. (A) Large size fraction (3-100 µm): *Haptophyta* (top), *Dinoflagellata* (middle), and *Bacillariophyta* (bottom), showing the 15 most abundant and unique ASVs per taxonomic group. (B) Small size fraction (0.2-3 µm): top 15 ASVs of primary producers. Bubble plots represent ASVs (18S rRNA V4-V5 region), with bubble size proportional to relative abundance per site, calculated within each size fraction [32]. Taxonomies were assigned using PR2 v5.1 at the most precise rank supported by the sequence [32]. ASVs were ordered using hierarchical clustering (Ward's method; ward.D). ASV feature IDs and taxon strings are given in Datasheet S5.

Table S1. Analysis of covariance (ANCOVA) of taxon-level transcript concentrations across regions and cruises.

phylum	region adjusted *P* value	cruise x region adjusted *P* value
Arthropoda	0.001*	0.761
Bacillariophyta	0.000*	0.130
Bolidophyceae	0.049*	0.761
Chlorophyta	0.000*	0.490
Chordata	0.034*	0.684
Ciliophora	0.007*	0.761
Cnidaria	0.108	0.415
Dictyochophyceae	0.007*	0.607
Dinoflagellata	0.000*	0.000*
Haptophyta	0.003*	0.712
MAST 3-4	0.039*	0.345
Pelagophyceae	0.003*	0.345

Gradients 1-3 transcripts of anabolic and catabolic genes as listed in Table S1 were included in the analysis. FDR-adjusted *P* value of < 0.05 are considered significantly different, as indicated by *

Table S2. Analysis of variance (ANOVA) of taxon-level TiP and relative transcript abundance between the Diel1 and G3-diel time series

phylum	TiP<br>adjusted *P* value	relative abundance<br>adjusted *P* value
Arthropoda	NA	0.008*
Bacillariophyta	0.000*	0.000*
Bolidophyceae	0.000*	0.000*
Chlorophyta	0.000*	0.000*
Chordata	0.020*	0.440
Ciliophora	NA	0.000*
Cnidaria	0.020*	0.958
Dictyochophyceae	0.000*	0.000*
Dinoflagellata	0.000*	0.000*
Haptophyta	0.000*	0.000*
MAST 3-4	0.000*	0.000*
Pelagophyceae	0.389	0.000*

FDR-adjusted *P* value of < 0.05 are considered significantly different, as indicated by *. NA indicates insufficient non-zero entries.

Table S3. PERMANOVA (adonis2) testing for differences in Bray-Curtis community composition among three regions (Gyre, STZ, NTZ) within cruise

Cruise	Df	R^2	*F*	*P* value
G1	2	0.1866	3.3217	0.002
G2	2	0.2364	8.2041	0.001
G3	2	0.1749	12.298	0.001

Factor tested is region (Gyre, STZ, NTZ). Df indicates degrees of freedom for the factor. R^2 represents the proportion of variance explained. *F* is the pseudo-*F* statistic. *P*-value is permutation-based (999 permutations). Analyses were conducted using the top 15 ASVs.

Supplemental Datasheet S1

Kofam annotations [40] representing anabolic genes required for primary production (photosynthesis, carbon fixation, light harvesting, and chlorophyll and carotenoid biosynthesis), and respiration/catabolic genes required for the TCA cycle, oxidative phosphorylation, and the degradation of carbohydrates, lipids, proteins, amino acids, and fatty acids. Kofams with ambiguous pathway assignments were excluded.

Datasheet fields:

KO: KEGG Orthology identifier

definition: KO definition

ko_pathway: KEGG pathway map identifier

name: KEGG pathway name

class: KEGG pathway class

trophic_cat: TiP category assigned for this study

Supplemental Datasheet S2

Mean transcript concentrations (10^6 transcripts L^{-1}) for the G1, G2, G3, and combined G1–G3 datasets. All samples and size classes were included. Taxa are sorted by mean transcript concentration in descending order. The top 12 taxonomic groups selected for this study are indicated by a shaded grey box. NA stands for Not Available. Dinoflagellata transcript concentrations were not scaled.

Datasheet fields:

phylum-class: Lowest common ancestor (LCA)-annotated phylum- or class-level taxonomy

mean_G1_million_transcripts_per_L: Mean transcript concentration across samples from the G1 transect (10^6 transcripts L^{-1})

mean_G2_million_transcripts_per_L: Mean transcript concentration across samples from the G2 transect (10^6 transcripts L^{-1})

mean_G3_million_transcripts_per_L: Mean transcript concentration across samples from the G3 transect (10^6 transcripts L^{-1})

mean_G1_G3: Mean transcript concentration across samples from the G1, G2, and G3 transects combined (10^6 transcripts L^{-1})

rank_transcript_concentrations: Rank based on mean transcript concentration

Supplemental Datasheet S3

Relative transcript abundance (proportion) of genus-level annotated transcripts within each phylum/class for the G1, G2, G3, and combined G1–G3 datasets. Genera are sorted by proportion in descending order. Only the top 12 taxonomic groups (phylum/class level) selected for this study are listed. NA stands for Not Available.

Datasheet fields:

phylum-class: Lowest common ancestor (LCA)-annotated phylum- or class-level taxonomy

genus: Lowest common ancestor (LCA)-annotated genus-level taxonomy

G1_within_phylum_ratio : Relative transcript abundance of the genus within its phylum/class across samples from the G1 transect

G2_within_phylum_ratio : Relative transcript abundance of the genus within its phylum/class across samples from the G2 transect

G3_within_phylum_ratio : Relative transcript abundance of the genus within its phylum/class across samples from the G3 transect

G1_G2_G3_within_phylum_ratio : Relative transcript abundance of the genus within its phylum/class across samples from the combined G1, G2, and G3 transects

Supplemental Datasheet S4

Mean ASV relative abundances for the top 12 taxonomic groups featured in this study. Relative abundances were aggregated by PR2 taxonomic strings, and mean relative abundances were calculated separately for each transect (G1–G3) and size fraction (0.2–3 µm and 3–100 µm). An overall mean relative abundance across all datasets was also calculated, with missing values (NA) treated as zero. The underlying ASV datasets are described in Key et al. (2026) [32].

Datasheet fields:

phylum_class: Phylum- or class-level taxonomy using NCBI nomenclature

PR2_taxonString: Full taxonomic classification string assigned using the PR2 database

NCBI_taxid: Closest matching NCBI Taxonomy identifier.

0.2um_G1: Mean relative abundance in the small size fraction (0.2–3 µm) across all samples from Gradient 1 (G1)

3um_G1: Mean relative abundance in the large size fraction (3–100 µm) across all samples from Gradient 1 (G1)

0.2um_G2: Mean relative abundance in the small size fraction (0.2–3 µm) across all samples from Gradient 2 (G2)

3um_G2: Mean relative abundance in the large size fraction (3–100 µm) across all samples from Gradient 2 (G2)

0.2um_G3: Mean relative abundance in the small size fraction (0.2–3 µm) across all samples from Gradient 3 (G3)

3um_G3: Mean relative abundance in the large size fraction (3–100 µm) across all samples from Gradient 3 (G3)

mean (NA == 0): Mean relative abundance across all transects and size fractions, with missing values (NA) treated as zero

Supplemental Datasheet S5

ASV feature IDs and taxon strings corresponding to Figures 6, S15, and S16. ASVs are listed in the order they appear in the figures. The datasets have been described in Key et al., 2026 [32].

Datasheet fields:

cruise: Dataset from which the data were obtained (e.g., Gradients 1 (G1), Gradients 2 (G2), Gradients 3 (G3))

size fraction: Filter size fraction from which data were obtained (e.g., small: 0.2-3 μm; large: 3-200 μm for G1, 3-100 μm for G2 and G3)

plot_order: Numeric order of the ASVs corresponding to the figures

phylum_class: Phylum/class level taxonomy

featureID: Unique ASV ID for reference in the database

ASVname_short: Abbreviated taxonomy name as depicted in the figures

taxonString: Full taxonomic classification string assigned using the PR2 database
